# Spatial PK–PD mismatch in tumor-targeting antibodies and ADCs: from lesion access to pharmacologic execution

**DOI:** 10.3389/fonc.2026.1952445

**Published:** 2026-09-22

**Authors:** Sitong Lai, Shanshan Li

**Affiliations:** 1 School of Pharmaceutical Sciences, Sun Yat-sen University, Guangzhou, China; 2 Dongzhimen Hospital, Beijing University of Chinese Medicine, Beijing, China

**Keywords:** antibody-drug conjugate, ImmunoPET, lesion heterogeneity, monoclonal antibody, pharmacokinetics, spatial pharmacology, target engagement, tumor penetration

## Abstract

Systemic pharmacokinetics and target expression do not show whether tumor cells have received enough antibody to produce the intended effect. Tumor-targeting antibodies must reach individual lesions, cross tumor tissue, bind accessible targets, and, for some platforms, align with intracellular or neighboring-cell processes required for action. This critical narrative review organizes evidence by where exposure and effect become discordant. We define four spatial PK–PD mismatches (1): systemic exposure without adequate lesion access (2); lesion uptake without adequate microscopic distribution; (3) target expression without sufficient *in vivo* engagement; and (4) engagement without spatially appropriate pharmacologic execution. Biological resistance after adequate spatial drug exposure is treated separately. We synthesize direct human distribution studies, lesion-resolved exposure–outcome studies, target-heterogeneity studies, preclinical interventions, and mechanistic models in a five-tier evidence hierarchy. Human immunoPET, fluorescence window studies, quantitative tissue imaging, and single-cell spatial pharmacobiology show substantial heterogeneity across lesions and cells, whereas lesion-level exposure–outcome evidence remains limited. Prior work has also shown that distribution effects depend on dose, affinity, antigen density, internalization, linker–payload properties, and bystander activity; the present review does not treat penetration as a universal determinant of efficacy. We therefore propose a mismatch-directed development workflow in which the suspected limiting step determines the measurement, corrective intervention, and validation endpoint. Spatial PK–PD is best viewed as a mechanism-based development framework rather than a validated routine companion diagnostic.

## 1 Introduction: target positivity is not pharmacologic coverage

Target expression is central to oncology drug development, but it does not establish that an administered antibody reaches and treats the relevant tumor cells. A circulating antibody must reach each clinically relevant lesion, cross the vascular interface, move through heterogeneous interstitium, bind an accessible epitope, and remain at sufficient concentration for long enough to complete its mechanism. Early models of macromolecular transport already separated vascular exchange, interstitial convection and diffusion, binding, internalization, and metabolism (1–6). The practical question is therefore not only whether a tumor expresses the target, but whether the drug achieves adequate pharmacologic coverage of the cells or compartments that must be controlled.

Common exposure measurements answer different questions. Plasma concentration establishes systemic availability, not lesion access. Whole-lesion PET measures regional signal but does not by itself resolve blood-pool activity, nonspecific retention, microscopic gradients, or cell-level occupancy. Tumor homogenates average exposed and unexposed regions, while immunohistochemistry identifies antigen in sampled tissue without showing whether the therapeutic epitope is accessible in the current lesion. Even engagement does not guarantee execution: receptor-blocking or Fc-active antibodies and ADCs have different downstream requirements, and ADCs additionally depend on productive internalization, trafficking, payload release, and sufficient local payload exposure (7, 8).

The resulting discordance is visible clinically. HER2-targeted imaging can identify substantial lesion-to-lesion variability and occasionally detect target-positive metastases that were not represented by the primary tumor specimen, but false-positive and discordant findings also occur (9–14). Tissue studies using labeled antibodies have shown that tumors with comparable target expression can differ in total accumulation and in the depth and uniformity of microscopic distribution (15–17). More recently, single-cell spatial pharmacobiology has directly demonstrated that drug delivery and target engagement vary among cells and are shaped by stromal neighborhoods in human solid tumors (18). These observations make a simple target-positive/target-negative model inadequate, but they do not yet justify the opposite overstatement that spatial imaging has become a validated predictor of clinical benefit.

Multiscale distribution is not a new concept. Prior transport, imaging, and modeling studies have already shown that antibody distribution depends on dose, affinity, antigen density, tissue architecture, and internalization; ADC studies further integrate linker–payload behavior and bystander activity. A recent review framed spatial pharmacology broadly around anti-cancer immunomodulators, tumor–immune organization, spatial omics, and target accessibility (19), while contemporary ADC reviews cover construct design, internalization, payload action, biomarkers, and resistance (20, 21). The contribution of the present review is therefore not to propose penetration as a new or universal explanation of efficacy. The review instead uses the location of exposure–effect discordance as its organizing unit. It separates four spatial mismatch classes from post-exposure biological resistance, grades evidence by the strength of inference it supports, and links each mismatch to a corresponding measurement, corrective strategy, and validation endpoint.

The framework is applied mainly to tumor-cell-targeting monoclonal antibodies and ADCs; T-cell engagers are used as a boundary example. Its unit of analysis is the point at which adequate upstream exposure fails to produce the next required pharmacologic step. The four mismatches are systemic exposure without lesion access, lesion uptake without microscopic distribution, target expression without effective engagement, and engagement without spatially aligned execution. Direct human distribution studies, lesion-level exposure–outcome associations, target-heterogeneity studies, preclinical interventions, and models are treated as distinct evidence types rather than as interchangeable support.

## 2 Literature approach and scope

This is a critical narrative review and was not designed as a systematic review. PubMed, Embase, and Web of Science were searched through 27 July 2026 using combinations of antibody therapeutics, monoclonal antibody, antibody–drug conjugate, tumor penetration, intratumoral distribution, target engagement, receptor occupancy, binding-site barrier, immunoPET, fluorescence-guided surgery, spatial proteomics, spatial pharmacobiology, bystander effect, and PK–PD modeling. Google Scholar citation chaining was used to recover foundational transport studies and human window-of-opportunity investigations. ClinicalTrials.gov was searched to verify relevant imaging and tissue-pharmacology study designs. Targeted searches through 27 August 2026 were used to identify newly published literature directly relevant to spatial pharmacology, ADC quality attributes and bioanalysis, intracellular trafficking, and organoid models. Priority was given to original human imaging or tissue studies, mechanistic experiments with direct spatial readouts, and quantitative models connecting systemic exposure with lesion-, tissue-, or cell-scale processes. The strategy supports a mechanistic synthesis and should not be interpreted as exhaustive evidence identification. ChatGPT (OpenAI; GPT-5.6 Sol model) was used after evidence extraction and citation verification to assist with manuscript drafting, language refinement, structural editing, and figure-legend wording. All AI-assisted wording was reviewed by the authors. Scientific claims and references were checked against the primary literature, and evidence tables and figures were independently reviewed for accuracy. The authors retain full responsibility for the final content.

The core scope includes antibodies directed against tumor-cell surface antigens and ADCs administered systemically for solid tumors. Studies of antibody fragments and engineered formats are included when they isolate the effects of molecular size, affinity, avidity, or dose on spatial disposition. Immune-checkpoint antibodies, radioimmunoconjugates, and T-cell-engaging bispecifics are used as boundary examples rather than parallel therapeutic literatures. The review does not catalogue approved products, summarize every ADC trial, or treat all molecular mechanisms of acquired resistance as spatial pharmacology. Post-delivery resistance is included only where it must be distinguished from inadequate delivery or engagement.

## 3 Definitions, boundaries, and evidence hierarchy

Spatial PK–PD describes how drug exposure and pharmacologic effect relate across nested anatomical scales. Whole-body clearance and normal-organ disposition determine the amount available to tumors. Lesion perfusion, permeability, anatomical site, size, necrosis, and target burden shape regional uptake. Within a lesion, margins, cores, perivascular niches, fibrotic stroma, viable nests, and necrotic regions can receive different concentrations. At the cellular level, distance from vessels, local antigen density, epitope accessibility, internalization, and neighboring cells further modify exposure (22–25). Averaging at one scale can therefore conceal failure at the next.

Terminology requires narrow operational definitions. Systemic exposure is concentration over time in plasma or blood. Lesion accumulation is total drug or imaging signal within a defined lesion. Penetration is movement away from the vascular or tissue-entry boundary, whereas microscopic distribution is the concentration pattern across tissue regions. Target accessibility is the fraction of target molecules reachable by the construct. Target engagement is actual *in vivo* drug–target binding, and receptor occupancy is the proportion of available receptors bound at a defined time (8). Endocytosis refers to uptake of a surface-bound target–ADC complex into intracellular vesicles. Intracellular trafficking begins after uptake and includes endosomal sorting, recycling, movement toward late endosomes or lysosomes, degradation, linker processing, and payload escape or export. Endocytosis therefore does not establish productive payload release (26, 27). Pharmacodynamic activity requires evidence that the intended downstream process occurred. Key operational definitions distinguishing commonly conflated spatial‑PK‑PD terms are summarized in **Table 1**.

**Table 1:** Terms that should not be used interchangeably in spatial PK–PD.

Term	Operational definition	Preferred measurement	Common misinterpretation
Systemic exposure	Drug concentration over time in plasma or blood	Plasma PK; total antibody, conjugated antibody, and payload assays for ADCs	Adequate plasma exposure proves adequate tumor exposure
Lesion accumulation	Total drug or imaging signal within a defined lesion	ImmunoPET/SPECT; quantitative specimen imaging; tissue concentration	Avidity or bulk concentration equals target occupancy
Penetration	Movement away from the vascular or tissue-entry boundary	Distance-to-vessel analysis; whole-slide fluorescence; autoradiography	Higher total uptake necessarily means deeper penetration
Microscopic distribution	Spatial pattern of drug concentration across tissue compartments	Quantitative histology; multiplex imaging; spatial proteomics	A tumor homogenate represents all viable regions
Target accessibility	Fraction of target physically reachable by the administered construct	Therapeutic-antibody imaging; epitope-specific tissue assay	Pathology positivity establishes *in vivo* accessibility
Target engagement	Binding of administered drug to its intended target *in vivo*	Competitive/occupancy assay; calibrated drug–target colocalization	Lesion uptake or proximity automatically equals occupancy
Endocytosis	Uptake of a surface-bound target–ADC complex from the plasma membrane into intracellular vesicles	Time-resolved uptake assay; imaging or quantitation of internalized ADC	Internalization proves lysosomal delivery or payload release
Intracellular trafficking	Post-endocytic sorting through recycling, endosomal, lysosomal, or other routes that determine ADC processing and payload release	Compartment-resolved colocalization; trafficking markers; released-payload and PD assays	Lysosomal proximity alone proves active-payload exposure
Spatial execution	Alignment of engaged drug with effector cells, productive intracellular compartments, or susceptible neighbors	Paired PD biopsy; spatial effector analysis; intracellular/payload mapping	Any post-binding resistance is a spatial failure
Pharmacologic coverage	Working construct describing the fraction and topology of relevant cells completing the modality-specific exposure-to-action sequence	Integrated multiscale measurement; no universal assay	A single scalar biomarker is already validated

Pharmacologic coverage refers here to the proportion and spatial distribution of relevant tumor cells or microenvironmental compartments that receive enough drug to complete the modality-specific sequence from engagement to downstream action. The required coverage is mechanism-dependent. Receptor blockers may require broad membrane occupancy, whereas Fc-mediated activity also requires competent effector cells in the relevant location. Non-bystander ADCs may require direct delivery to most vulnerable target-positive cells. Bystander-capable ADCs can tolerate some antigen-negative cells but remain constrained by payload release, diffusion, potency, and local metabolism. Pharmacologic coverage is therefore an organizing construct, not a single biomarker or imaging threshold.

The distinction also prevents an important conceptual error: spatial underexposure and target heterogeneity are related but not identical. A target-negative region cannot bind the antibody even if delivery is excellent. A target-positive region may remain pharmacologically untreated because the antibody never reaches it. Conversely, an imaging tracer may accumulate in a lesion through vascular or nonspecific processes without engaging enough target-positive cells. Studies that relate heterogeneous HER2 expression to response demonstrate the clinical relevance of target composition, but they do not by themselves establish delivery as the mediating mechanism (28–32). A spatial PK–PD framework must preserve these distinctions rather than re-label all heterogeneity as penetration failure.

To prevent evidence of one type from being used to support a stronger claim, the review applies five tiers. Tier 1 comprises direct human studies in which an administered antibody or closely related labeled construct was measured at the lesion, tissue, or cellular level. Tier 2 comprises human lesion-resolved exposure–outcome studies. Tier 3 includes human target-heterogeneity–outcome associations without direct measurement of administered-drug distribution. Tier 4 includes preclinical interventions with a spatial exposure or pharmacodynamic readout. Tier 5 includes mechanistic experiments and mathematical models. The hierarchy is not a quality score: a rigorous Tier 5 model may be essential for hypothesis generation, but it cannot by itself establish clinical predictive validity. A five‑tier evidence hierarchy is presented in **Table 2** to grade the strength of human and preclinical observations.

**Table 2:** Evidence hierarchy used in this review.

Tier	Definition	What it can support	Principal limitation
Tier 1	Direct human administered-drug distribution or engagement	Existence, scale, and pattern of human spatial exposure	Often small, selected cohorts; single dose/lesion/time
Tier 2	Human lesion-resolved exposure–outcome evidence	Association between uptake pattern and lesion/patient outcome	Does not prove that correcting uptake restores benefit
Tier 3	Human target-heterogeneity–outcome evidence without drug-distribution measurement	Clinical relevance of target topology	Cannot distinguish target absence, delivery failure, or downstream resistance
Tier 4	Preclinical intervention with spatial exposure or PD readout	Mechanistic modifiability and causal hypothesis generation	Translation depends on human dose, architecture, and toxicology
Tier 5	Mechanistic experiment or mathematical model	Parameter sensitivity and falsifiable prediction	Cannot establish human exposure or predictive validity alone

## 4 A four-mismatch framework for spatial pharmacologic failure

The four mismatches are sequential but can coexist within the same patient or lesion. One metastasis may have adequate access while another does not; an avid lesion may still contain perivascular underexposure; a target-positive cell may remain unengaged; and an engaged cell may lack the spatial conditions required for execution. Biological resistance despite an adequate spatial drug challenge is considered separately because its explanation and corrective strategy differ from those of underexposure. Observable patterns, underlying mechanisms, preferred measurement modalities, and current evidence maturity for each spatial mismatch are outlined in **Table 3**. The sequential framework of four spatial PK‑PD mismatches and post‑exposure biological resistance is illustrated in **Figure 1**.

**Table 3:** Spatial PK–PD mismatches: observable patterns, measurements, and evidence maturity.

Mismatch	Observable pattern	Mechanisms within scope	Preferred measurement	Strongest current evidence
Systemic-to-lesion access	Adequate plasma PK; low or heterogeneous lesion uptake	Perfusion, organ barriers, lesion architecture, antigen sink, target-mediated disposition	Plasma PK plus whole-body immunoPET; current-lesion biopsy	Tier 2 for selected drug–target pairs
Bulk uptake-to-microscopic distribution	Avid lesion or adequate bulk concentration; peripheral/perivascular localization	Binding-site barrier, antigen density/affinity, internalization, ECM/CAF exclusion, low mobility	Whole-specimen imaging; vessel-distance analysis; spatial proteomics	Tier 1 human tissue; Tier 4 intervention
Target expression-to-engagement	Positive IHC/ISH; weak therapeutic-antibody binding or discordant *in vivo* imaging	Sampling, temporal evolution, therapeutic-epitope mismatch, masking, shedding, inaccessible topology	Current-lesion pathology; therapeutic-antibody imaging; calibrated colocalization/occupancy	Tier 1 for selected targets
Spatially contingent engagement-to-execution	Drug bound; required effector cell, productive compartment, or susceptible neighbor not aligned	Effector exclusion, nonproductive spatial trafficking, insufficient payload field, failed multicellular complex	Paired PD biopsy; spatial effector/trafficking/payload analysis	Predominantly Tier 4–5; limited Tier 1
Boundary: post-exposure biological resistance	Adequate spatial challenge; absent response	Target-independent signaling, payload-target alteration, efflux, DNA repair, anti-apoptotic adaptation	Confirm preceding spatial steps, then mechanism-specific resistance assays	Outside the core spatial framework

**Figure 1:**
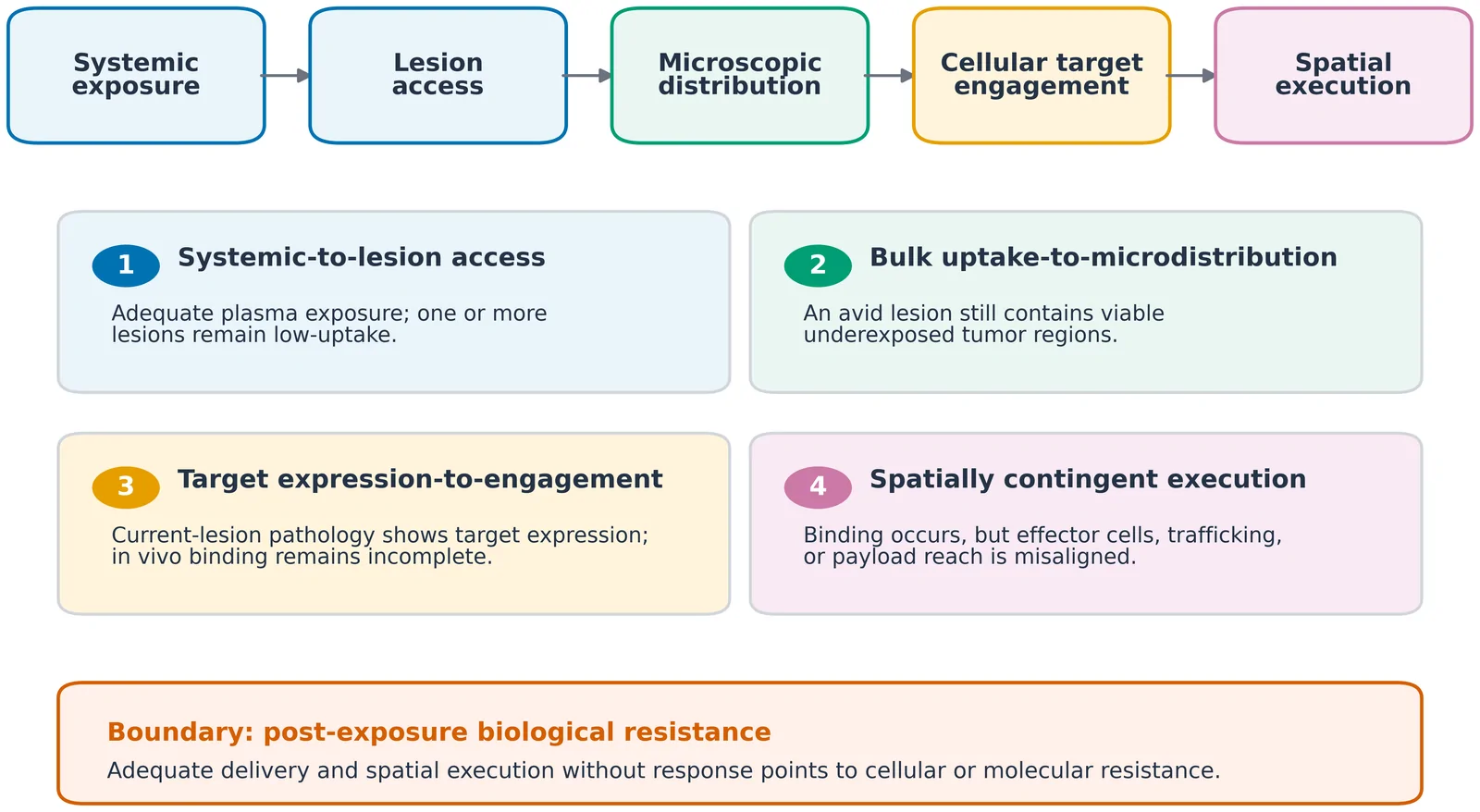
Four spatial PK–PD mismatches and the boundary of post-exposure biological resistance. The upper sequence follows an administered antibody from systemic exposure through lesion access, microscopic distribution, cellular target engagement, and spatial execution. The four mismatch classes identify the first pharmacologic step that is inadequate. Persistent failure after adequate delivery, engagement, and spatial execution is classified separately as post-exposure biological resistance.

### 4.1 Systemic-to-lesion access mismatch

Systemic-to-lesion access mismatch occurs when plasma exposure is compatible with activity but one or more tumor deposits receive insufficient drug. Antibodies distribute slowly and incompletely from blood into many solid tumors. Abnormal vessels are heterogeneously perfused and permeable, intratumoral lymphatics are limited, and elevated interstitial fluid pressure can reduce convective transport (1, 3, 4, 23). These features vary by lesion. Anatomical site, lesion size, necrosis, vascular phenotype, and stromal structure can therefore produce regional underexposure despite an apparently adequate plasma AUC.

Target-mediated disposition adds another source of mismatch. Soluble antigen, normal-tissue target, circulating target-positive cells, and highly perfused target-rich lesions can consume antibody before it reaches less accessible deposits. Rapid turnover or internalization may further increase local sink behavior (33, 34). These mechanisms can produce nonlinear systemic PK and unequal lesion supply. Dose selection must therefore consider the exposure needed to sustain unbound antibody across clinically important lesions, not merely detectable plasma concentrations.

ImmunoPET is particularly valuable at this mismatch because it surveys the whole body. Early A33 studies established that radiolabeled antibodies could quantify tumor localization and reveal variable uptake in colorectal cancer (15, 35, 36). HER2-targeted PET subsequently demonstrated heterogeneous uptake among metastases and showed that imaging can sometimes identify lesions whose target status differs from archived tissue (9, 10, 12, 13). Yet PET avidity is not target occupancy. Signal depends on delivery, blood-pool clearance, tracer mass, radionuclide residualization, internalization, nonspecific retention, lesion size, and partial-volume effects. False-positive findings in biopsy-verified studies are a direct warning against converting a lesion-level signal into a binary molecular diagnosis without context (10, 11).

Lesion-level outcome studies move this mismatch closer to clinical relevance. In ZEPHIR, baseline 89Zr-trastuzumab PET showed marked interlesional heterogeneity among patients receiving T-DM1; a negative or mixed HER2-PET pattern was associated with early treatment failure, and combining HER2-PET with early FDG-PET improved response classification (14). A pilot 64Cu-DOTA-trastuzumab study similarly examined whether baseline uptake could predict T-DM1 outcome (37). These investigations are Tier 2 evidence: they link *in vivo* antibody uptake to outcome, but they do not prove that increasing uptake would restore benefit. Low uptake may reflect inadequate delivery, inaccessible target, altered target biology, or several processes simultaneously.

The corrective strategy depends on the cause. Increasing dose or adding a loading dose may overcome systemic sinks and supply poorly perfused lesions, but only if toxicity and normal-tissue binding permit. Altering infusion interval may improve time above a lesion-access threshold without raising peak concentration. A smaller construct can cross vascular and interstitial barriers more rapidly but may clear too quickly to sustain exposure. Vascular normalization can transiently improve perfusion and pressure gradients, although excessive antiangiogenic treatment can prune vessels and reduce delivery (38–40). Thus, lesion access should be treated as a dynamic exposure problem, not as an intrinsic binary property of a tumor.

### 4.2 Bulk uptake-to-microscopic distribution mismatch

A lesion can accumulate substantial antibody while viable regions remain underexposed. This occurs when drug entering the lesion is captured near vessels or the tissue boundary faster than it can move inward. The binding-site barrier describes this interaction: high-affinity binding to abundant antigen can create steep perivascular gradients, so strong capture by proximal cells reduces delivery to more distant cells (7, 41). The gradient becomes more pronounced with rapid internalization, high antigen density, low administered molar dose, or long diffusion distances.

Affinity therefore has a context-dependent, non-monotonic relation with useful distribution. Very high-affinity constructs can localize strongly but remain near vessels, whereas lower-affinity constructs may penetrate farther at the cost of retention (42–44). Rapid internalization can deepen a proximal sink by removing surface-bound antibody and sustaining an inward concentration gradient (45). Quantitative models accordingly predict penetration from the joint effects of dose, affinity, antigen concentration, internalization, permeability, and time (46, 47).

Human studies confirm that bulk uptake and microscopic distribution can diverge. Quantitative A33 studies showed substantial tumor uptake with heterogeneous, time-dependent microdistribution (15, 16). In 24 patients with head and neck cancer, panitumumab fluorescence varied across patients, tumors, and intratumoral regions; larger tumors tended to show lower concentration and greater peripheral-to-central disparity despite EGFR expression (17). Coadministration of unlabeled antibody also altered penetration of a labeled conjugate in human tumors (48). The observed distribution effects should therefore be interpreted in the context of antigen density, binding, dose, internalization, and therapeutic mechanism rather than as an independent effect of penetration. These studies establish that dosing can modify microscopic distribution in patients, while clinical benefit from that modification remains to be demonstrated.

Single-cell spatial pharmacobiology adds cellular and stromal resolution. In human head and neck and pancreatic tumors receiving panitumumab–IRDye800, delivery and target engagement varied markedly among cells, and periostin-rich matrix assemblies and FAP-positive fibroblast neighborhoods were associated with lower antibody delivery (18). The study links measured drug signal to defined spatial neighborhoods rather than to a nonspecific notion of ‘stroma.’ Whether the same associations hold across other targets, therapeutic doses, disease stages, and repeated dosing remains to be tested.

Diagnosing this mismatch therefore requires paired measurements. A PET value or homogenate concentration should be interpreted with whole-slide or spatial imaging when microscopic coverage is the question. Candidate interventions include dose or loading-mass adjustment, parent-antibody coadministration, affinity tuning, smaller formats, and vascular or stromal modulation. Their effects are context-dependent: parent antibody can reduce ADC occupancy at proximal receptors, smaller formats may lose cumulative exposure, and broader penetration may add little when the mechanism already generates an adequate bystander field. The objective is mechanism-specific coverage within the therapeutic window, not maximal penetration.

### 4.3 Target expression-to-engagement mismatch

Target expression-to-engagement mismatch occurs when a diagnostic assay indicates target positivity but the therapeutic molecule does not bind enough relevant cells *in vivo*. Sampling is an immediate source of discordance: a biopsy represents one region and may predate treatment by months or years, whereas systemic therapy acts across evolving metastases. HER2 studies document intratumoral and interlesional heterogeneity and show that target-directed imaging can identify lesions not predicted by archived tissue (10, 13, 28, 29). Biopsy–imaging discordance may reflect biological evolution, sampling error, assay thresholds, delivery, or nonspecific tracer retention; it should not be treated as proof that either modality is intrinsically definitive.

Diagnostic and therapeutic epitopes can also differ. Standard pathology measures target abundance with an assay antibody, whereas therapeutic accessibility may be altered by steric hindrance, receptor conformation, ligand occupancy, heterodimerization, shedding, or spatial clustering. Target turnover and internalization further determine whether binding produces sustained surface occupancy or rapid sequestration (33, 34). Thus, antigen positivity does not guarantee *in vivo* engagement, and antibodies directed at the same receptor can differ in accessibility.

ImmunoPET partly bridges this gap because the tracer is derived from the therapeutic antibody or a closely related construct. It assesses accessible binding sites across the body under *in vivo* delivery conditions, but each voxel still integrates specific binding with vascular and interstitial activity. Antibody mass is critical: too little tracer can be sequestered by normal organs or proximal target, while added unlabeled antibody can alter biodistribution and contrast (9, 12). Imaging protocols should therefore be treated as pharmacologic experiments with defined mass and timing. Dedicated clinical methods and anti-HER3 studies show how immunoPET can contribute to engagement assessment (49, 50).

Tissue colocalization of administered drug with its target is more specific but remains limited to the sampled time and region. Window studies can identify target-positive, drug-low areas, and single-cell approaches can relate local target abundance, drug signal, and stromal context (18). Even these measurements do not establish receptor occupancy or functional blockade unless they are calibrated against available receptor and linked to downstream pharmacodynamics. A robust interpretation therefore triangulates current-lesion pathology, *in vivo* imaging, and tissue drug–target analysis rather than treating them as interchangeable tests.

### 4.4 Spatially contingent engagement-to-execution mismatch

Spatially contingent engagement-to-execution mismatch begins after the antibody reaches and binds its target. The drug can be correctly localized yet fail because the downstream machinery is not colocated with the engaged cell or relevant compartment. Fc-active antibodies, for example, depend on the presence and competence of natural killer cells, macrophages, complement, and other effectors near antibody-coated tumor cells. By contrast, loss of downstream target dependence despite adequate blockade belongs to post-exposure biological resistance.

ADCs add intracellular and neighborhood-scale requirements. Binding is followed by endocytosis, but cellular uptake alone is not sufficient. Productive execution requires intracellular sorting to compartments that support antibody catabolism or linker cleavage, followed by generation of active payload and access to its intracellular target. Recycling or nondegradative sequestration can therefore produce ADC-positive but payload-poor cells (26, 27). These processes also interact with tissue distribution. Rapid internalization may increase payload delivery to proximal cells while strengthening a perivascular sink, whereas slower uptake may broaden antibody distribution but reduce intracellular payload flux. Construct-level ADC reviews describe these dependencies (20).

Bystander activity extends this process beyond the target-positive cell. Membrane-permeable payloads can move to neighboring cells and partly compensate for heterogeneous antigen expression or direct ADC distribution (51–54). The effective radius remains finite and depends on release, permeability, extracellular binding, metabolism, diffusion, and recipient-cell susceptibility. Dual-label studies show that antibody and payload can diverge spatially after uptake and release (55). Bystander capability therefore changes the required topology of direct ADC delivery; it does not remove the need to measure where active payload reaches susceptible cells.

The same biomarker consequently carries different spatial implications across platforms. T-DM1 has limited bystander reach and may require direct ADC access to a large fraction of vulnerable HER2-positive cells. Topoisomerase-I ADCs can bridge some antigen-negative gaps, yet still require enough target-positive producer cells in the correct neighborhoods. In a prospective neoadjuvant T-DM1 plus pertuzumab study, none of the 16 evaluable heterogeneous tumors achieved pathological complete response compared with 55% of nonheterogeneous tumors (30). More homogeneous HER2 expression has also been associated with better trastuzumab deruxtecan outcomes in gastric cancer (32). These are Tier 3 associations because drug distribution and payload fields were not measured directly.

### 4.5 Post-exposure biological resistance: outside the core spatial framework

Post-exposure biological resistance is outside the core spatial framework. This category applies when sufficient drug reaches the lesion, engages the relevant cells, and completes the required spatial execution, yet the tumor persists because the biological mechanism is no longer effective. Examples include target-independent signaling after adequate receptor blockade, payload-target alteration, drug efflux, enhanced DNA repair, anti-apoptotic adaptation, and intrinsic insensitivity of recipient cells. Lysosomal or trafficking abnormalities also belong here when the ADC reaches the appropriate cell and the defect is cell-intrinsic rather than a failure of spatial delivery to the productive compartment.

This boundary prevents the framework from absorbing all antibody or ADC resistance. It also changes the recommended intervention. A spatial mismatch may be addressed by dose, schedule, format, epitope, coadministration, or microenvironmental modulation. Confirmed post-exposure resistance usually requires a different target, payload, mechanism, or combination. The distinction cannot be inferred from nonresponse alone; it requires evidence that the preceding spatial steps were adequate.

## 5 Platform-specific modifiers of pharmacologic coverage

Spatial requirements are mechanism-specific. Tumor-targeting monoclonal antibodies, non-bystander ADCs, bystander-capable ADCs, and T-cell engagers do not require identical coverage. Deeper penetration may improve one construct and dilute productive occupancy for another. A high lesion signal can indicate accessible target, residualized catabolite, or nonspecific retention. Antigen heterogeneity can be decisive for a non-bystander ADC but partly bridged by a payload with sufficient local diffusion. **Table 4** summarizes these differences.

**Table 4:** Platform-specific requirements for pharmacologic coverage.

Platform	Minimum spatial requirement	Dominant spatial failure risks	Meaning of target heterogeneity	Most informative measurements
Tumor-targeting monoclonal antibody	Sufficient breadth and duration of occupancy; effector co-location when Fc activity matters	Lesion underexposure, perivascular capture, incomplete occupancy, effector exclusion	Target-negative cells remain unblocked; indirect effects may partly compensate	Plasma PK, immunoPET, occupancy/PD biopsy, spatial immune context
Non-bystander ADC	Direct delivery, endocytosis, productive intracellular trafficking, and payload release in most vulnerable target-positive cells	Low tolerated molar dose, binding-site barrier, heterogeneous target, nonproductive trafficking	Target-negative cells receive little payload; heterogeneity is strongly limiting	Intact ADC distribution, target colocalization, endocytosis/trafficking, released-payload and spatial PD assays
Bystander-capable ADC	Network of target-positive producer cells plus payload diffusion to susceptible neighbors	Insufficient producer-cell coverage, limited payload radius, recipient-cell resistance	Some negative gaps can be bridged; benefit depends on topology and payload properties	Dual-label antibody/payload imaging, target topology, spatial PD radius
T-cell engager (boundary case)	Co-location of drug, antigen-positive tumor cell, CD3-positive effector, and productive synapse	Effector exclusion, nonproductive saturation, target loss, cytokine-limited dosing	Negative cells may escape unless secondary immune effects operate	Tumor/T-cell distribution, ternary-complex modeling, spatial activation markers

### 5.1 Tumor-targeting monoclonal antibodies

For receptor-blocking antibodies, the relevant quantity is the duration and breadth of occupancy required to suppress signaling in the tumor cell population that sustains growth. A shallow but saturating distribution may be effective in a small, highly vascular lesion yet leave the core of a larger lesion untreated. If Fc-dependent cytotoxicity contributes substantially, the spatial distribution of Fcγ-receptor-bearing cells and their activation state becomes part of the pharmacodynamic system. The antibody does not act on an isolated receptor; it creates an interface between tumor cells, immune cells, complement, and the extracellular environment.

Affinity–distribution relationships are also context-dependent. High affinity can improve retention at low concentration but restrict penetration, whereas lower affinity can broaden distribution while reducing occupancy or residence time (42, 45). Bivalency, receptor clustering, antigen density, dose, and internalization modify that trade-off. Human A33 studies illustrate sustained uptake and measurable microdistribution (15, 16, 35), but they are best interpreted as one target-specific example within a broader transport framework rather than as a general rule for all antibody–target pairs.

### 5.2 Antibody–drug conjugates

ADCs are particularly vulnerable to spatial PK–PD mismatch because their tolerated antibody molar dose is constrained by payload toxicity. A naked antibody can sometimes be dosed high enough to saturate proximal antigen and drive deeper penetration; an ADC may reach its systemic toxicity ceiling before achieving comparable molar exposure. The binding-site barrier is therefore not a theoretical curiosity for ADCs. Multiscale models and direct imaging show that tissue distribution, internalization, intracellular processing, and payload movement must be considered together (56, 57).

Coadministration of unconjugated parent antibody has been studied as a context-dependent saturation strategy. By occupying proximal antigen, the parent antibody can allow a smaller fraction of ADC to penetrate more deeply. Preclinical experiments and quantitative models show that the effect depends on antigen density, ADC dose, and bystander activity and can be favorable, neutral, or unfavorable under different conditions (58–60). In human fluorescence studies, co-administered unlabeled antibody changed microscopic distribution of a labeled antibody conjugate (48). The efficacy implications are conditional on antigen density, ADC dose, bystander activity, and the specific saturation or competitive strategy. The human data demonstrate pharmacologic modifiability, but not yet an improved therapeutic index from routine parent-antibody coadministration.

Smaller ADC formats offer another route. Increased diffusivity and faster tissue equilibration can improve single-cell targeting and antitumor activity in models, but faster renal or systemic clearance and altered payload disposition can narrow exposure time (61). Likewise, a high drug-to-antibody ratio increases payload delivered per binding event but may increase hydrophobicity, aggregation, clearance, and normal-tissue toxicity. Spatial performance is therefore an emergent property of the complete construct, not a feature that can be optimized by changing one component in isolation.

Bystander-capable payloads reduce—but do not abolish—the need for direct ADC coverage. Their effective radius depends on the concentration released by target-positive cells, membrane permeability, extracellular binding, and susceptibility of neighboring cells. Three-dimensional imaging demonstrates a continuum rather than a binary classification of bystander versus non-bystander payloads (53). Models similarly predict that payload exposure distant from vessels is sensitive to diffusivity and dose and may respond only weakly to the proportion of antigen-positive cells under some conditions (57). The relevant question is whether the local payload field spans the spatial gaps present in a given tumor without producing unacceptable normal-tissue exposure. Contemporary reviews reinforce the need to treat bystander effect as a graded, construct-specific property rather than a binary label (21, 54).

### 5.3 Boundary case: T-cell-engaging antibodies

T-cell-engaging bispecific antibodies expose the limitations of a one-target occupancy model. Productive activity requires simultaneous or sequential colocalization of drug, tumor antigen, CD3, and a viable effector cell. Very high drug concentrations can reduce formation of productive ternary complexes through independent saturation of each cell, and the relationship between exposure and synapse formation can be non-monotonic. Tumor target density, CD3 affinity, effector-to-target ratio, tissue distribution, and cytokine release must therefore be modeled together (62). These agents are not a main focus of this review, but they make an important conceptual point: the pharmacologically relevant unit may be a spatially organized multicellular complex rather than an occupied receptor.

## 6 Measuring spatial PK–PD in patients

No single method spans whole-body distribution, microscopic delivery, molecular engagement, and downstream effect. The appropriate measurement is determined by the suspected mismatch. Plasma PK is necessary for interpreting all other measurements but insufficient for locating failure. ImmunoPET offers whole-body lesion mapping. Fluorescence window studies and autoradiography resolve tissue distribution in resected specimens. Spatial proteomics can link drug signal, target, and cell neighborhoods. Paired biopsies can assess pharmacodynamic execution. Multiscale models connect these measurements but cannot substitute for them. **Figure 2** aligns measurement scale with the evidence claim each method can support.

**Figure 2:**
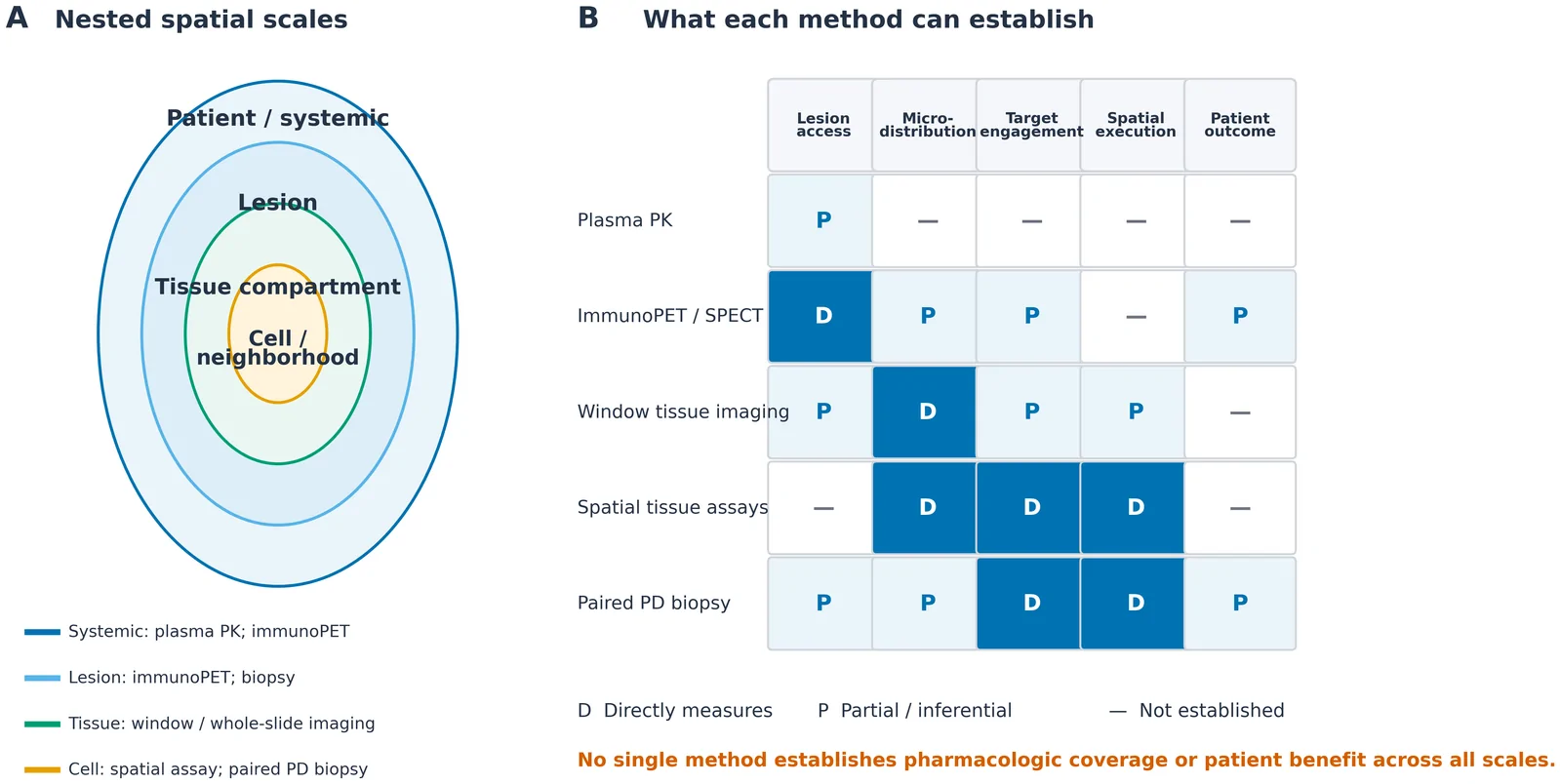
Spatial scale determines which component of antibody PK–PD is observable. **(A)** Four nested scales link patient-level exposure to lesion, tissue-compartment, and cell/neighborhood measurements. **(B)** Capability matrix showing whether commonly used methods directly measure (D), partially or inferentially assess (P), or do not establish (—) lesion access, microscopic distribution, target engagement, spatial execution, or patient outcome. For ADCs, immunoPET and fluorescence imaging generally report the labeled antibody or conjugate and do not directly measure local free active payload concentration or intracellular release efficiency. No single method provides a complete estimate of pharmacologic coverage.

### 6.1 Plasma pharmacokinetics

Plasma PK establishes whether the patient received and retained systemic drug exposure. It identifies accelerated clearance, immunogenicity, target-mediated nonlinearity, and interpatient variability. For ADCs, separate assays for conjugated antibody, total antibody, and released payload can reveal deconjugation and systemic payload exposure. These measurements are indispensable for dose selection and safety, but a conventional central-and-peripheral compartment model does not establish concentration in a specific lesion or cell. A normal plasma profile should therefore narrow the diagnosis toward lesion access or downstream mismatch, not close the investigation.

### 6.2 ImmunoPET and SPECT

Radiolabeled antibodies can map accessible whole-body distribution over several days. The physical half-life of zirconium-89 is compatible with intact antibody kinetics, and initial trials established feasible imaging of diverse therapeutic targets (63). Trastuzumab studies demonstrated lesion-level HER2 imaging and clinically relevant heterogeneity (9, 10, 12, 13). Anti-HER3 immunoPET illustrated how the same platform can be used to assess target engagement during early drug development (50). PD-1 and PD-L1 imaging provides a boundary example in which uptake reflects the distribution of both target and immune context, and exploratory studies have associated uptake with response (12, 64, 65).

The signal requires careful interpretation. Antibody mass changes clearance and target saturation. Residualizing radionuclides can remain after catabolism, so tumor activity may outlast intact functional antibody. Small lesions are affected by partial-volume error; blood volume, necrosis, and nonspecific irreversible uptake alter regional values. Recent quantitative analyses show that tissue-to-plasma ratios and correction for antibody catabolism may be necessary to separate specific uptake from residualized background (66, 67). Harmonized acquisition and kinetic interpretation remain active methodological problems (68). ImmunoPET is consequently a lesion-access and accessible-target assay whose target engagement and predictive meaning must be validated for each drug–tracer context (49, 69).

For ADC development, immunoPET can reduce uncertainty in target selection, reveal normal-organ sinks, and support dose or antibody-mass selection before broad clinical exposure to a payload-bearing construct (70, 71). The signal primarily follows the radiolabeled antibody backbone or a closely related tracer; it does not directly quantify intracellular release efficiency, local concentrations of free active payload, or the resulting bystander field. Component-specific bioanalysis of total antibody, conjugated ADC, free payload, and drug-to-antibody ratio is therefore complementary rather than interchangeable with spatial imaging (72). ZEPHIR illustrates both the value and the boundary: 89Zr-trastuzumab uptake contributed lesion-level information relevant to T-DM1 outcome, while the mechanism underlying low uptake required additional biological interpretation (14, 73).

### 6.3 Fluorescence window-of-opportunity studies

Fluorescence-labeled antibodies administered before surgery directly connect systemic dosing with resected tissue. Early cetuximab–IRDye800 studies established clinical feasibility and tumor specificity in head and neck cancer (74). Bevacizumab–IRDye800CW microdosing demonstrated that vascular-targeted fluorescence could quantify uptake in primary breast cancer (75). Panitumumab–IRDye800 studies subsequently measured tumor-size effects, peripheral–central gradients, and construct distribution in head and neck and pancreatic tumors (48, 76, 77). A small loading-dose study and later coadministration analyses showed that unlabeled antibody mass can alter tumor-specific uptake and microscopic distribution (48, 78).

These trials have structural limitations. Participants generally have resectable disease and may not represent heavily pretreated metastatic populations. The labeled construct must be shown to retain relevant binding and disposition. Imaging doses may be below therapeutic doses, while a therapeutic dose can partly saturate the barrier that the tracer is intended to measure. Tissue is sampled at one postoperative time point, which may miss earlier gradients or later redistribution. Fluorescence attenuation and tissue processing also require calibration. For ADCs, fluorescence usually follows the labeled antibody or conjugate rather than free active payload, so it should not be interpreted as a direct map of local payload concentration or intracellular release efficiency. These constraints define the questions that fluorescence studies can answer reliably.

### 6.4 Single-cell spatial pharmacobiology

Single-cell spatial pharmacobiology extends window studies by measuring the administered drug, target, cell identity, and microenvironmental architecture in the same tissue coordinate system. The 2026 study using panitumumab–IRDye800 in human head and neck and pancreatic tumors identified marked variability in drug delivery and engagement and associated low-delivery regions with periostin-rich matrix and FAP-positive fibroblast neighborhoods (18). This resolves a longstanding ambiguity in conventional histology: a collagen-rich region may correlate with low delivery, but multiplex spatial analysis can determine whether the association persists within specific cellular neighborhoods and across tumors.

The method remains a development platform. Signal calibration to absolute antibody concentration and occupancy is challenging; tissue processing can alter epitopes and fluorescence; the number of tumors and targets studied is still limited. Association with a stromal neighborhood does not prove that removing that neighborhood will improve therapeutic outcome. Most importantly, single-cell delivery at a single time point cannot by itself establish cumulative exposure or patient-level efficacy. Its immediate contribution is mechanistic stratification: it can identify whether a target-positive cell was reached and which local features accompany failure.

### 6.5 Paired pharmacodynamic biopsies and multiscale models

Paired biopsies are required when the suspected problem is execution rather than access. For a receptor-blocking antibody, appropriate markers may include receptor phosphorylation and downstream signaling. For an ADC, internalized antibody, endosomal or lysosomal localization, released-payload measurements, and downstream markers such as DNA damage, mitotic arrest, or apoptosis represent different steps and should not be treated as interchangeable evidence. The biopsy time must match the kinetic sequence; a late sample can miss transient engagement, while an early sample can precede cell death. Spatial co-registration is preferable to separate bulk assays because it identifies whether pharmacodynamic activity occurred in drug-exposed target-positive cells rather than in a different compartment.

Multiscale models connect plasma, vascular, interstitial, cellular, and intracellular processes that cannot be sampled continuously in patients. Foundational models described how perfusion, diffusion, binding, and metabolism generate spatial gradients (5, 6). More recent ADC models incorporate realistic vascular geometry, internalization, payload release, and bystander diffusion (56, 57). Their value lies in falsifiable prediction—for example, identifying when dose, affinity, or parent-antibody coadministration should alter distribution. A model becomes clinically useful only after parameters are anchored to human measurements and predictions are prospectively tested. It should not be used to manufacture tissue-level certainty from plasma data alone.

## 7 Human evidence map: what has actually been demonstrated?

Human evidence is strongest for heterogeneous access and distribution, more limited for direct target engagement, and still developing for exposure–response causality. The five-tier hierarchy prevents target heterogeneity, imaging uptake, and therapeutic response from being treated as interchangeable observations. **Table 5** summarizes Tier 1 studies that directly measured an administered antibody or related tracer and Tier 2 studies that linked lesion uptake with response. **Table 6** separates Tier 3 target-heterogeneity outcome studies in which administered-drug distribution was not measured.

**Table 5:** Direct human administered-drug studies and lesion-resolved exposure–outcome evidence (Tiers 1–2).

Study/tier	Setting and evaluable sample	Administered construct, mass, and timing	Scale/method	Principal finding and boundary
(35)/Tier 1	Metastatic colorectal cancer with hepatic metastases; N = 20; localization evaluable in all (selective in 19/20)	Radiolabeled target-directed antibody; not a clinically administered therapeutic analogue. 131I-mAb A33, 2–5 mCi and 0.2–50 mg protein, IV; 7–8 days before surgery.	Whole-body imaging, lesion localization, and quantitative tissue analysis	Selective but variable tumor localization; no cell-level target-occupancy measurement
(16)/Tier 1	Colorectal carcinoma; N = 12	Radiolabeled humanized target-directed antibody; resection substudy of the A33 phase I program. 131I-huA33; phase I protein-dose range 0.25–10 mg/m², IV; 7 days before surgery.	Macroscopic and microscopic autoradiography with kinetic tissue analysis	Heterogeneous and time-dependent microdistribution; radioactivity does not prove intact-antibody occupancy
(9)/Tier 1	Metastatic HER2-positive breast cancer; N = 14	Radiolabeled therapeutic analogue of trastuzumab. 37 MBq 89Zr-trastuzumab; 10 or 50 mg in trastuzumab-naive patients and 10 mg in patients receiving trastuzumab; PET on days 2–5; optimal visualization on days 4–5.	Whole-body immunoPET and lesion uptake quantification	Marked interlesional heterogeneity; tracer mass and residualizing signal influence interpretation
(74)/Tier 1	Head and neck squamous cell carcinoma; N = 12	Fluorescently labeled therapeutic analogue of cetuximab. Cetuximab–IRDye800, 2.5, 25, or 62.5 mg/m², IV; Infusion on day 0; surgery on day 3.	*In vivo*, intraoperative, specimen, and tissue fluorescence imaging	Feasible tumor-selective imaging across scales; early safety/specificity study without efficacy testing
(78)/Tier 1	Head and neck squamous cell carcinoma; N = 6 (3 per preload cohort)	Fluorescently labeled therapeutic analogue with unlabeled parent-antibody preload. Unlabeled cetuximab 10 or 100 mg, followed 1 h later by cetuximab–IRDye800CW 25 mg/m², IV; Surgery 3–4 days after labeled-antibody infusion.	Plasma PK, *in vivo* optical imaging, and specimen fluorescence	The 100-mg preload increased circulating labeled antibody, tumor fluorescence, and tumor-to-background ratio; very small cohort
(50)/Tier 1	Advanced HER3-positive solid tumors; N = 6	Radiolabeled therapeutic analogue of anti-HER3 antibody GSK2849330. 37 MBq 89Zr-GSK2849330; baseline total mass 8 or 24 mg; repeat study after graded total masses from 24 mg to 30 mg/kg; PET on days 2 and 5; repeat administration approximately 2 weeks later.	Whole-body immunoPET with target-engagement and mass-saturation modeling	Demonstrated imaging-supported antibody-mass selection and target saturation; six-patient development study
(48)/Tier 1	Resectable head and neck squamous cell carcinoma; 24 enrolled; key microdistribution comparison n=22 (12 loading; 10 non-loading)	Fluorescently labeled therapeutic analogue with or without unlabeled panitumumab. Loading cohort: 100 mg panitumumab then 0.5 or 1 mg/kg panitumumab–IRDye800CW; non-loading cohort: 25 or 50 mg labeled antibody; total antibody 0.3–2.6 mg/kg; 1–5 days before surgery.	Quantitative tumor concentration, whole-slide fluorescence, and vessel-distance analysis	Coadministration broadened microscopic distribution without increasing normal-tissue uptake; no therapeutic-outcome endpoint
(18)/Tier 1	HNSCC, PDAC, and NSCLC; 50 enrolled; bulk quantification n=43; single-cell spatial cohort n=30	Fluorescently labeled therapeutic analogue of panitumumab at subtherapeutic imaging doses. HNSCC: 50 mg; PDAC: 25/50/75 mg with 100 mg unlabeled panitumumab or 50 mg alone; NSCLC: 25/50/75 mg; HNSCC/NSCLC 1–5 days and PDAC 2–5 days before surgery.	Bulk tumor quantification integrated with single-cell drug imaging and high-plex spatial proteomics	Directly mapped heterogeneous cellular delivery and engagement; periostin-rich ECM and FAP-positive CAF neighborhoods were associated with low delivery
(14, 73)/Tier 2	Advanced HER2-positive breast cancer; parent ZEPHIR cohort: 60 enrolled, 56 analyzed; imaging–transcriptomic substudy: 24 matched biopsied metastases	Radiolabeled therapeutic analogue used to characterize accessible HER2 before T-DM1. Parent protocol: 37 MBq (± 10%) 89Zr-trastuzumab plus 50 mg unlabeled trastuzumab; T-DM1 3.6 mg/kg every 3 weeks; baseline HER2-PET and early FDG-PET.	Whole-body lesion-resolved HER2-PET integrated with early FDG-PET, treatment outcome, and matched-metastasis transcriptomics	Negative or heterogeneous uptake identified high early-failure risk; low-uptake/nonresponding lesions were enriched for extracellular-matrix and hypoxia-related programs. Associations were nonrandomized and do not prove that correcting uptake improves outcome.
(37)/Tier 2	Metastatic HER2-positive breast cancer; N = 10	Radiolabeled therapeutic analogue used before T-DM1. 45 mg unlabeled trastuzumab followed by 5 mg 64Cu-DOTA-trastuzumab; T-DM1 3.6 mg/kg every 3 weeks; PET at 21–25 and 47–49 h after tracer, before T-DM1.	Lesion-resolved PET uptake associated with time to treatment failure	Baseline uptake showed exploratory association with T-DM1 outcome; ten-patient pilot

Full dosing, timing, and construct-relation fields are provided in **Supplementary Table S1**.

**Table 6:** Human target-heterogeneity outcome studies without direct drug-distribution measurement (Tier 3).

Study/tier	Setting	Therapy/target assessment	Outcome association	Why evidence is indirect for spatial PK–PD
(28)/Tier 3	HER2-positive metastatic breast cancer	Trastuzumab; intratumoral HER2 heterogeneity	Heterogeneity associated with response and survival	Administered-drug distribution was not measured
(29)/Tier 3	HER2-positive gastric cancer	Trastuzumab; intratumoral HER2 heterogeneity	Heterogeneity associated with reduced efficacy	Cannot separate target-negative clones from delivery or downstream resistance
(30)/Tier 3	Early HER2-positive breast cancer; 164 treated, 157 evaluable	Neoadjuvant T-DM1 plus pertuzumab; HER2 heterogeneity	0/16 heterogeneous tumors achieved pCR versus 55% of nonheterogeneous tumors	Strong target-topology association without direct drug-distribution measurement
(31)/Tier 3	HER2-positive endometrial cancer	Trastuzumab/T-DM1 context; genetic HER2 heterogeneity	Heterogeneity associated with resistance	Biological and spatial mechanisms remain entangled
(32)/Tier 3	HER2-positive gastric cancer	Trastuzumab deruxtecan; intratumoral HER2 heterogeneity	More homogeneous expression associated with better response/outcomes	Observational target architecture; payload field not measured

Tier 1 evidence began with quantitative radioantibody studies showing measurable and variable tumor localization, followed by tissue autoradiography, immunoPET, fluorescence window studies, and cellular spatial imaging (9, 15–18, 35, 36, 48, 74, 75). These studies establish that the first three mismatch components can be interrogated in selected settings. No single modality measures the full sequence, and most tissue studies represent one dose, one lesion, and one time point.

Tier 2 evidence remains uncommon. ZEPHIR linked baseline 89Zr-trastuzumab patterns and early metabolic response with T-DM1 outcome (14). Subsequent transcriptomic integration of biopsied ZEPHIR lesions associated low antibody uptake with extracellular-matrix and related biological programs, while nonresponding lesions showed hypoxia and matrix signatures (73). A pilot 64Cu-DOTA-trastuzumab study explored baseline uptake as a predictor of T-DM1 outcome (37). Exploratory PD-L1 and PD-1 immunoPET studies reported associations between tracer uptake and checkpoint-blockade response (12, 65). These studies support lesion-level biological validity and early outcome association; none establishes a universally applicable threshold or proves that correcting uptake changes outcome.

Tier 3 evidence demonstrates that target architecture is clinically consequential without isolating spatial drug delivery. HER2 heterogeneity has been associated with trastuzumab resistance, inferior T-DM1 pathological response, and poorer outcomes with HER2-directed therapy across breast, gastric, and endometrial cancer settings (28–32). A heterogeneous tumor may fail because target-negative clones are intrinsically untargetable, target-positive regions are underexposed, execution is spatially incomplete, or post-exposure biology is resistant. The evidence supports the importance of target topology but does not identify the limiting step by itself.

Several validation gaps remain. No single imaging threshold has been prospectively validated as adequate pharmacologic coverage across antibody or ADC platforms. Greater microscopic uniformity has not been shown prospectively to improve patient-level outcomes across constructs. Stromal signatures identified with one drug–target pair require independent validation before use as predictive biomarkers. A single pretreatment or surgical specimen also cannot represent cumulative exposure during repeated dosing. These gaps define priorities for future validation and limit generalization across constructs and settings.

## 8 Discussion: correcting spatial mismatch and translating the framework

### 8.1 Corrective strategies aligned to the limiting step

Corrective strategies should follow the diagnosed mismatch. Dose or loading mass can improve lesion supply and saturate proximal binding sites, yet may increase normal-tissue exposure. Lower affinity can broaden distribution while reducing retention. Smaller formats enter tissue rapidly but may clear before adequate cumulative exposure. A membrane-permeable payload can bridge antigen-negative gaps and simultaneously widen toxicity. Optimization therefore targets mechanism-specific coverage and therapeutic index, not one isolated distribution metric.

Dose is a structural determinant of spatial distribution. At low molar doses, high-affinity antibodies can be captured by the first accessible antigen. A loading dose or higher maintenance concentration can saturate proximal sites and drive drug farther into tissue (43, 47). Fractionation may sustain a concentration gradient while limiting peak toxicity, although repeated small doses can also be consumed by the same proximal sink. For ADCs, the optimal schedule depends on linker stability, payload half-life, target recycling, and normal-tissue recovery.

Parent-antibody coadministration is a targeted saturation strategy. Its appeal is greatest for high-density antigens and ADCs with limited bystander reach. Quantitative experiments show that benefit diminishes with lower antigen density or greater bystander activity (58). Transient competitive inhibitors offer a more controllable alternative by temporarily reducing binding during tissue entry, then allowing high-affinity engagement after distribution (59). These approaches require direct confirmation that deeper distribution compensates for lower ADC occupancy per proximal cell; otherwise, apparent improvement in uniformity can conceal reduced payload delivery to the most target-rich population.

Affinity should be tuned to the intended dose, antigen density, internalization rate, and lesion architecture. Fragment studies demonstrate that very high affinity can restrict penetration, whereas intermediate affinity can improve distribution without unacceptable loss of retention (42, 44). Bivalent avidity can preserve tumor retention while allowing weaker monovalent binding, but it also depends on receptor density and spacing. Epitope selection can alter internalization, receptor clustering, accessibility through glycocalyx, and competition with ligand.

Smaller formats—Fab, single-chain fragments, single-domain antibodies, and engineered scaffolds—diffuse faster and may access compact tissue regions more effectively. Their shorter half-life reduces normal-organ exposure but can also reduce total tumor exposure. Half-life extension restores exposure while partly sacrificing the kinetic advantage. The appropriate format therefore depends on whether failure is dominated by slow penetration, rapid clearance, or insufficient residence. Preclinical single-cell imaging showing improved distribution and efficacy with smaller ADC formats is encouraging, but human translation must account for different renal handling and payload toxicology (61).

ADC design and product quality jointly influence where intact conjugate travels and where active payload acts. Relevant attributes include drug-to-antibody ratio and its distribution, conjugation-site heterogeneity, aggregate or fragment content, residual unconjugated antibody, linker–payload stability, free-payload-related impurities, and functional potency. These attributes can alter clearance, target binding, premature payload release, tissue exposure, and toxicity; differences across lots, formulations, or development stages can therefore modify an observed PK–PD relationship. Manufacturing control, stability assessment, and fit-for-purpose release testing should document the quality attributes relevant to the clinical material (72, 79).

Bystander potential should be measured as a spatially graded, construct-specific property. Three-dimensional models show different depth and timing of pharmacodynamic effects across payload classes (53). Dual-label approaches can separate antibody and payload-associated signals in preclinical models, illustrating that their spatial distributions can diverge after internalization and release (55). However, label-derived payload signals are not automatically equivalent to free active payload, and current human spatial imaging rarely provides direct measurements of local payload concentration or intracellular release efficiency. A translational ADC assessment should therefore combine intact-ADC distribution with component-specific bioanalysis and pharmacodynamic readouts rather than infer payload exposure from antibody uptake alone (72).

Abnormal perfusion and elevated interstitial pressure are modifiable in principle. Judicious VEGF-pathway inhibition can transiently normalize vascular structure, improve pressure gradients, and increase drug penetration in preclinical models (38, 39). The key word is transient. Excessive or prolonged antiangiogenic therapy can reduce vessel density and drug delivery, so sequence and dose must be optimized rather than assumed. Clinical application would require a biomarker of the normalization window and direct evidence that the antibody reaches previously underexposed regions.

Stromal modulation is more uncertain. Enzymatic matrix remodeling, inhibition of matrix synthesis, fibroblast targeting, and reduction of solid stress may improve transport, but stroma can also restrain tumor spread and maintain tissue integrity. The association of periostin-rich matrix and FAP-positive fibroblast neighborhoods with low antibody delivery provides a rational starting point for intervention studies (18); it does not establish that broad fibroblast depletion is beneficial. The appropriate experiment is causal and spatial: perturb the candidate barrier, confirm improved drug distribution in the same compartment, and then determine whether pharmacodynamic activity and outcome improve without accelerating tumor behavior.

### 8.2 A mismatch-directed clinical-development workflow

A practical development program begins with the observed failure pattern. Low systemic exposure calls for investigation of clearance, immunogenicity, or target-mediated disposition. Adequate plasma exposure with non-avid lesions calls for whole-body imaging and current-lesion pathology. Avid lesions with peripheral pharmacodynamic response require tissue microdistribution analysis. Drug–target colocalization without mechanism-specific pharmacodynamics requires paired tissue analysis and a search for spatial execution failure or post-exposure resistance. **Figure 3** presents this sequence.

**Figure 3:**
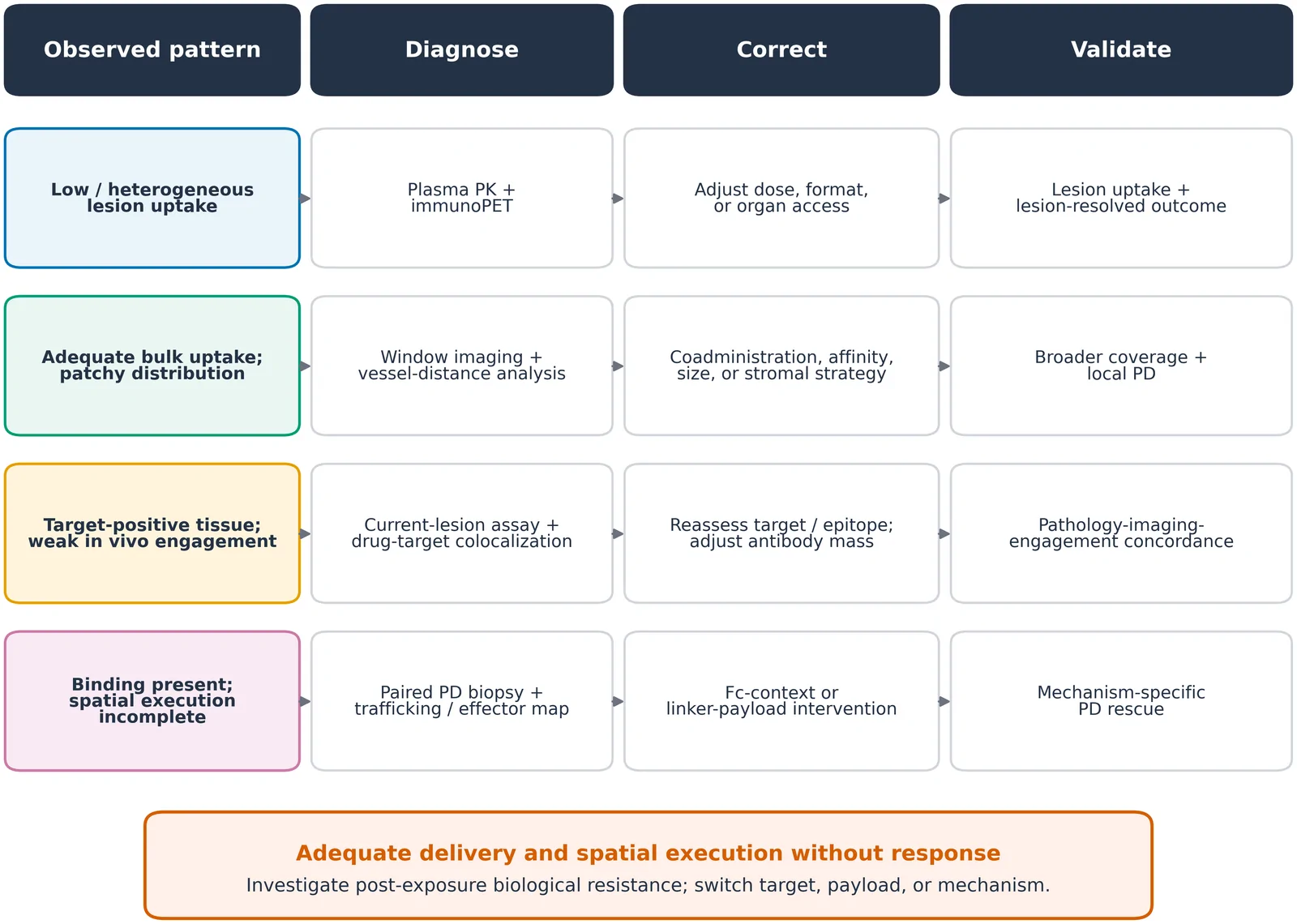
Mismatch-directed clinical-development workflow. The observed failure pattern determines the diagnostic method, corrective strategy, and validation endpoint. Low or heterogeneous lesion uptake prompts systemic PK and immunoPET assessment; adequate bulk uptake with patchy distribution requires tissue-scale analysis; target-positive tissue with weak *in vivo* engagement requires current-lesion and drug–target assessment; and binding without spatial execution requires paired pharmacodynamic and trafficking or effector mapping. Adequate delivery and spatial execution without response redirects investigation toward post-exposure biological resistance.

Early clinical trials should use dose-ranging to characterize distribution as well as toxicity. A tracer or fluorescent microdose can be administered with graded unlabeled antibody mass to determine whether target sinks and binding-site barriers change. Surgical-window studies can compare macroscopic accumulation, microscopic distribution, target engagement, and pharmacodynamic markers at prespecified times. In metastatic disease, lesion-level imaging should be linked to lesion-level response rather than only patient-level RECIST. This matters because a patient can be classified as having stable disease while individual lesions with low uptake progress and avid lesions regress.

Biomarker development requires a staged validation pathway. Analytical validity comes first: the drug or tracer signal must be reproducible and calibrated. Biological validity requires demonstration that the signal corresponds to the intended exposure or engagement process. Lesion-level association should then be tested against local pharmacodynamics and response. Predictive validity requires treatment interaction—showing that the biomarker identifies benefit from a specific therapy rather than prognosis alone. Finally, prospective utility must show that acting on the measurement improves decisions or outcomes. Most current spatial pharmacology measurements are between biological validity and early lesion-level association; they should not be described as routine predictive biomarkers.

Implementation also has practical costs. Radiolabeling can alter affinity, charge, aggregation, or clearance; PET requires radiochemistry, harmonized acquisition, reconstruction, and lesion quantification; fluorescence and spatial tissue assays require standardized sampling, processing, segmentation, and quality control. Serial imaging and biopsies can increase trial cost, operational complexity, development time, and participant burden. These constraints argue against comprehensive spatial profiling for every program. A staged, hypothesis-driven design is more realistic: begin with the measurement that tests the suspected limiting step, expand only if that result changes the development decision, and use less invasive longitudinal measures whenever they can answer the same question.

### 8.3 Controversies and interpretive limits

Penetration should be interpreted in the context of mechanism. Broader distribution may improve coverage for some constructs but can reduce retention or dilute productive occupancy for others. A potent bystander ADC may not require direct antibody exposure of every susceptible cell. Receptor blockers, Fc-active antibodies, non-bystander ADCs, and bystander-capable ADCs therefore require different spatial metrics and coverage thresholds.

Bulk imaging rarely quantifies target engagement on its own. ImmunoPET uptake contains contributions from delivery, blood volume, binding, internalization, catabolism, and nonspecific retention. Receptor occupancy requires competitive or mass-balance measurements and preferably tissue calibration (49, 66, 67). Fluorescence signal likewise should not be equated with intact functional antibody unless degradation and nonspecific components have been addressed.

Bystander killing can mitigate antigen heterogeneity only under defined conditions. Payload must be released in sufficient quantity, remain active, cross membranes, and diffuse far enough to reach antigen-negative cells. Recipient cells must retain the payload target and lack relevant resistance mechanisms. Preclinical data support local bystander killing and a spectrum of tissue penetration (51, 53), while clinical heterogeneity studies show that substantial target-negative fractions can still predict poor outcome (30, 32). Bystander activity changes tolerance for heterogeneity but does not remove the importance of target architecture.

Stromal barrier signatures also require drug- and context-specific validation. Similar fibroblast and matrix neighborhoods were associated with low delivery in two human tumor types (18), but different antibodies vary in charge, affinity, dose, Fc interactions, and internalization, and tumors vary in vascular and interstitial architecture. A barrier signature becomes actionable only if it reproducibly predicts low delivery across relevant drug–target pairs and if modifying it improves exposure and downstream pharmacodynamics.

Spatial underexposure is best described as a candidate pharmacologic mechanism of primary or regional treatment failure. Resistance implies persistence despite an adequate drug challenge. A target-positive cell that never receives drug has demonstrated delivery failure, not cellular resistance. Conversely, a lesion with adequate access, engagement, spatial execution, and no response belongs in the post-exposure biological-resistance category. The distinction is clinically material because the remedies diverge.

### 8.4 Future validation agenda

The first priority is prospective lesion-level validation. Studies should pair quantitative immunoPET or other whole-body imaging with lesion-specific response and, where feasible, biopsy. A lesion that is non-avid and progresses provides different evidence from a lesion that is avid, target-engaged, and still progresses. Analyses should account for lesion size, anatomical site, blood pool, and prior treatment. Patient-level averaging should be avoided when the biological hypothesis is regional underexposure.

Second, tracer-dose findings must be bridged to therapeutic-dose pharmacology. Dose-escalation window studies can determine whether microscopic gradients flatten as antibody mass increases and whether the distribution of a labeled fraction remains representative. For ADCs, parent-antibody coadministration and fractionation should be tested with direct measurements of intact ADC, payload, and pharmacodynamic effect rather than inferred from total fluorescence alone.

Third, spatial PK–PD should become longitudinal. Baseline target architecture may change after therapy, while vascular permeability, stromal composition, antigen density, and internalization can evolve during response and at progression. Repeated whole-body imaging, strategically timed biopsies, and minimally invasive circulating markers can be integrated in a model that distinguishes stable patient-specific traits from treatment-induced changes. A single resection specimen is a cross-section of a dynamic system.

Fourth, the four-mismatch framework requires cross-platform validation. The same study design should be applied to multiple antibody–target pairs and to ADCs with distinct linker–payload properties. This will determine which barriers are conserved and which are construct-specific. The framework will be useful only if it predicts when an intervention should work. Randomized or otherwise controlled studies of dose, coadministration, schedule, or microenvironmental modulation should therefore include spatial exposure as a mechanistic intermediate and pharmacodynamic response as a downstream endpoint.

Finally, computational integration should be constrained by experimentally measured quantities. Plasma PK, lesion imaging, digital pathology, single-cell spatial data, and lesion response can be combined in hierarchical or mechanistic models. The useful outputs are clinically interpretable quantities: the probability that a lesion is underexposed, the fraction of target-positive cells above an effective concentration, and the intervention most likely to correct the limiting step. Uncertainty should be propagated explicitly because each measurement observes only part of the sequence.

### 8.5 Limitations of this review

This critical narrative review was designed to provide an explanatory framework rather than exhaustive effect estimates. Direct human evidence remains concentrated in HER2-, EGFR-, and A33-directed constructs and perioperative tracer studies, often at microdose or subtherapeutic antibody mass and single time points. Surgical cohorts preferentially represent resectable disease, while unsampled metastases and longitudinal exposure remain incompletely characterized. ImmunoPET and fluorescent-antibody signals combine target-dependent and target-independent processes and do not directly measure free active payload or receptor occupancy without orthogonal validation. Two-dimensional cell systems and tumor organoids are valuable for controlled studies of endocytosis, trafficking, payload release, and bystander killing. However, they do not reproduce systemic clearance, vascular delivery, organ sinks, interstitial transport, or the full stromal and immune architecture of human tumors. Their findings should therefore be treated as mechanistic evidence rather than direct estimates of *in vivo* spatial PK–PD (80). In addition, serial imaging, biopsies, radiochemistry, and high-dimensional tissue assays can increase cost, development time, operational complexity, and patient burden, which limits routine deployment and supports staged use. We did not conduct a formal risk-of-bias assessment or meta-analysis. The mismatch classes therefore remain a development framework requiring prospective, cross-platform validation.

## 9 Conclusion

Precision oncology has largely asked whether a tumor expresses a therapeutic target. For tumor-targeting antibodies and ADCs, target positivity and systemic exposure are upstream prerequisites; neither demonstrates that a clinically relevant cell population has experienced an adequate drug challenge. That challenge extends through lesion access, microscopic distribution, cellular engagement, and spatially aligned pharmacologic execution.

The proposed framework localizes failure to four spatial mismatch classes and separates them from post-exposure biological resistance. Human imaging and tissue studies can measure selected components of lesion access, microdistribution, and engagement, while Tier 2 studies such as ZEPHIR provide early evidence that lesion-resolved uptake patterns carry outcome information. The evidence that correcting these patterns improves patient-level benefit remains limited. Spatial PK–PD should therefore guide mechanism-based development: identify the suspected limiting step, select a measurement at the appropriate scale, test a targeted intervention, and validate the effect at lesion and patient levels. Its immediate contribution is a more exact account of why a target-positive tumor did—or did not—experience an adequate pharmacologic challenge.

## References

[1] Jain RK . Transport of molecules across tumor vasculature. Cancer Metastasis Rev. (1987) 6:559–93. doi: 10.1007/bf00047468 3327633

[2] Jain RK . Transport of molecules in the tumor interstitium: a review. Cancer Res. (1987) 47:3039–51. 3555767

[3] Baxter LT Jain RK . Transport of fluid and macromolecules in tumors. I. Role of interstitial pressure and convection. Microvasc Res. (1989) 37:77–104. doi: 10.1016/0026-2862(89)90074-5 2646512

[4] Baxter LT Jain RK . Transport of fluid and macromolecules in tumors. II. Role of heterogeneous perfusion and lymphatics. Microvasc Res. (1990) 40:246–63. doi: 10.1016/0026-2862(90)90023-k 2250603

[5] Baxter LT Jain RK . Transport of fluid and macromolecules in tumors. III. Role of binding and metabolism. Microvasc Res. (1991) 41:5–23. doi: 10.1016/0026-2862(91)90003-t 2051954

[6] Baxter LT Jain RK . Transport of fluid and macromolecules in tumors. IV. A microscopic model of the perivascular distribution. Microvasc Res. (1991) 41:252–72. doi: 10.1016/0026-2862(91)90026-8 2051960

[7] Weinstein JN van Osdol W . The macroscopic and microscopic pharmacology of monoclonal antibodies. Int J Immunopharmacol. (1992) 14:457–63. doi: 10.1016/0192-0561(92)90176-l 1618598

[8] Bartelink IH Jones EF Shahidi-Latham SK Lee PRE Zheng Y Vicini P . Tumor drug penetration measurements could be the neglected piece of the personalized cancer treatment puzzle. Clin Pharmacol Ther. (2019) 106:148–63. doi: 10.1002/cpt.1211 30107040 PMC6617978

[9] Dijkers EC Oude Munnink TH Kosterink JG Brouwers AH Jager PL de Jong JR . Biodistribution of 89Zr-trastuzumab and PET imaging of HER2-positive lesions in patients with metastatic breast cancer. Clin Pharmacol Ther. (2010) 87:586–92. doi: 10.1038/clpt.2010.12 20357763

[10] Ulaner GA Hyman DM Ross DS Corben A Chandarlapaty S Goldfarb S . Detection of HER2-positive metastases in patients with HER2-negative primary breast cancer using 89Zr-trastuzumab PET/CT. J Nucl Med. (2016) 57:1523–8. doi: 10.2967/jnumed.115.172031 27151988 PMC5050126

[11] Dehdashti F Wu N Bose R Naughton MJ Ma CX Marquez-Nostra BV . Evaluation of [(89)Zr]trastuzumab-PET/CT in differentiating HER2-positive from HER2-negative breast cancer. Breast Cancer Res Treat. (2018) 169:523–30. doi: 10.1007/s10549-018-4696-z 29442264 PMC5955803

[12] Bensch F Brouwers AH Lub-de Hooge MN de Jong JR van der Vegt B Sleijfer S . (89)Zr-trastuzumab PET supports clinical decision making in breast cancer patients, when HER2 status cannot be determined by standard work up. Eur J Nucl Med Mol Imaging. (2018) 45:2300–6. doi: 10.1007/s00259-018-4099-8 30058029 PMC6208812

[13] Ulaner GA Carrasquillo JA Riedl CC Yeh R Hatzoglou V Ross DS . Identification of HER2-positive metastases in patients with HER2-negative primary breast cancer by using HER2-targeted (89)Zr-pertuzumab PET/CT. Radiology. (2020) 296:370–8. doi: 10.1148/radiol.2020192828 32515679 PMC7543717

[14] Gebhart G Lamberts LE Wimana Z Garcia C Emonts P Ameye L . Molecular imaging as a tool to investigate heterogeneity of advanced HER2-positive breast cancer and to predict patient outcome under trastuzumab emtansine (T-DM1): the ZEPHIR trial. Ann Oncol. (2016) 27:619–24. doi: 10.1093/annonc/mdv577 26598545

[15] Scott AM Lee FT Jones R Hopkins W MacGregor D Cebon JS . A phase I trial of humanized monoclonal antibody A33 in patients with colorectal carcinoma: biodistribution, pharmacokinetics, and quantitative tumor uptake. Clin Cancer Res. (2005) 11:4810–7. doi: 10.1158/1078-0432.ccr-04-2329 16000578

[16] Ciprotti M Chong G Gan HK Chan A Murone C MacGregor D . Quantitative intratumoural microdistribution and kinetics of (131)I-huA33 antibody in patients with colorectal carcinoma. EJNMMI Res. (2014) 4:22. doi: 10.1186/s13550-014-0022-x 24995151 PMC4070025

[17] Lu G Fakurnejad S Martin BA van den Berg NS van Keulen S Nishio N . Predicting therapeutic antibody delivery into human head and neck cancers. Clin Cancer Res. (2020) 26:2582–94. doi: 10.1158/1078-0432.ccr-19-3717 31980465 PMC9398035

[18] Lu G Hickey JW Haist M Qin X Zhao E Naveed A . Single-cell spatial pharmacobiology identifies conserved stromal barriers to therapeutic antibody delivery in human solid tumors. Nat Biotechnol. (2026). doi: 10.1038/s41587-026-03152-x 42236958 PMC13390730

[19] Chen S . Spatial pharmacology of anti-cancer immunomodulators: a comprehensive review. Front Pharmacol. (2026) 17:1898418. doi: 10.3389/fphar.2026.1898418 PMC13543664 42699460

[20] Cheung A Liu Y Chenoweth AM Montaseri H Esapa B Chudasama V . Antibody-drug conjugate design and mechanisms of action for cancer treatment: state of the art and beyond. Physiol Rev. (2026) 106:1681–756. doi: 10.1152/physrev.00039.2025 41855081

[21] Lei L Zheng YB Tang SC . Antibody-drug conjugates in cancer therapy: do we need biomarkers? A comprehensive review. Cancer Metastasis Rev. (2026) 45:36. doi: 10.1007/s10555-026-10344-z 42185658 PMC13201299

[22] Dvorak HF Nagy JA Dvorak AM . Structure of solid tumors and their vasculature: implications for therapy with monoclonal antibodies. Cancer Cells. (1991) 3:77–85. 1711364

[23] Jain RK . Physiological barriers to delivery of monoclonal antibodies and other macromolecules in tumors. Cancer Res. (1990) 50:814s–9s. 2404582

[24] Minchinton AI Tannock IF . Drug penetration in solid tumours. Nat Rev Cancer. (2006) 6:583–92. doi: 10.1038/nrc1893 16862189

[25] Thurber GM Schmidt MM Wittrup KD . Antibody tumor penetration: transport opposed by systemic and antigen-mediated clearance. Adv Drug Delivery Rev. (2008) 60:1421–34. doi: 10.1016/j.addr.2008.04.012 18541331 PMC2820307

[26] Hammood M Craig AW Leyton JV . Impact of endocytosis mechanisms for the receptors targeted by the currently approved antibody-drug conjugates (ADCs)-a necessity for future ADC research and development. Pharm (Basel). (2021) 14:674. doi: 10.3390/ph14070674 34358100 PMC8308841

[27] Leyton JV . The endosomal-lysosomal system in ADC design and cancer therapy. Expert Opin Biol Ther. (2023) 23:1067–76. doi: 10.1080/14712598.2023.2285996 37978880

[28] Lee HJ Seo AN Kim EJ Jang MH Suh KJ Ryu HS . HER2 heterogeneity affects trastuzumab responses and survival in patients with HER2-positive metastatic breast cancer. Am J Clin Pathol. (2014) 142:755–66. doi: 10.1309/ajcpirl4guvgk3yx 25389328

[29] Wakatsuki T Yamamoto N Sano T Chin K Kawachi H Takahari D . Clinical impact of intratumoral HER2 heterogeneity on trastuzumab efficacy in patients with HER2-positive gastric cancer. J Gastroenterol. (2018) 53:1186–95. doi: 10.1007/s00535-018-1464-0 29633013 PMC6209002

[30] Filho OM Viale G Stein S Trippa L Yardley DA Mayer IA . Impact of HER2 heterogeneity on treatment response of early-stage HER2-positive breast cancer: phase II neoadjuvant clinical trial of T-DM1 combined with pertuzumab. Cancer Discov. (2021) 11:2474–87. doi: 10.1158/2159-8290.cd-20-1557 33941592 PMC8598376

[31] Shen S Ma W Brown D Da Cruz Paula A Zhou Q Iaosonos A . HER2 genetic intratumor heterogeneity is associated with resistance to trastuzumab and trastuzumab emtansine therapy in recurrent high-grade endometrial cancer. Mod Pathol. (2023) 36:100299. doi: 10.1016/j.modpat.2023.100299 37558129 PMC10841308

[32] Yoshino K Takamatsu M Ooki A Chin K Takahari D Osumi H . Clinical impact of intratumoral HER2 heterogeneity on trastuzumab deruxtecan efficacy in patients with HER2-positive gastric cancer. Gastric Cancer. (2026) 29:597–610. doi: 10.1007/s10120-026-01736-9 41925805 PMC13124857

[33] Ackerman ME Pawlowski D Wittrup KD . Effect of antigen turnover rate and expression level on antibody penetration into tumor spheroids. Mol Cancer Ther. (2008) 7:2233–40. doi: 10.1158/1535-7163.mct-08-0067 18645032 PMC2831054

[34] Schmidt MM Thurber GM Wittrup KD . Kinetics of anti-carcinoembryonic antigen antibody internalization: effects of affinity, bivalency, and stability. Cancer Immunol Immunother. (2008) 57:1879–90. doi: 10.1007/s00262-008-0518-1 18408925 PMC2840397

[35] Welt S Divgi CR Real FX Yeh SD Garin-Chesa P Finstad CL . Quantitative analysis of antibody localization in human metastatic colon cancer: a phase I study of monoclonal antibody A33. J Clin Oncol. (1990) 8:1894–906. doi: 10.1200/jco.1990.8.11.1894 2230877

[36] Carrasquillo JA Pandit-Taskar N O'Donoghue JA Humm JL Zanzonico P Smith-Jones PM . (124)I-huA33 antibody PET of colorectal cancer. J Nucl Med. (2011) 52:1173–80. 10.2967/jnumed.110.086165 PMC3394182 21764796

[37] Mortimer JE Bading JR Frankel PH Carroll MI Yuan Y Park JM . Use of (64)Cu-DOTA-trastuzumab PET to predict response and outcome of patients receiving trastuzumab emtansine for metastatic breast cancer: a pilot study. J Nucl Med. (2022) 63:1145–8. doi: 10.2967/jnumed.121.262940 34857660 PMC9364339

[38] Tong RT Boucher Y Kozin SV Winkler F Hicklin DJ Jain RK . Vascular normalization by vascular endothelial growth factor receptor 2 blockade induces a pressure gradient across the vasculature and improves drug penetration in tumors. Cancer Res. (2004) 64:3731–6. doi: 10.1158/0008-5472.can-04-0074 15172975

[39] Jain RK . Normalization of tumor vasculature: an emerging concept in antiangiogenic therapy. Science. (2005) 307:58–62. doi: 10.1126/science.1104819 15637262

[40] Goel S Duda DG Xu L Munn LL Boucher Y Fukumura D . Normalization of the vasculature for treatment of cancer and other diseases. Physiol Rev. (2011) 91:1071–121. doi: 10.1152/physrev.00038.2010 21742796 PMC3258432

[41] Fujimori K Covell DG Fletcher JE Weinstein JN . A modeling analysis of monoclonal antibody percolation through tumors: a binding-site barrier. J Nucl Med. (1990) 31:1191–8. 2362198

[42] Adams GP Schier R McCall AM Simmons HH Horak EM Alpaugh RK . High affinity restricts the localization and tumor penetration of single-chain fv antibody molecules. Cancer Res. (2001) 61:4750–5. 11406547

[43] Graff CP Wittrup KD . Theoretical analysis of antibody targeting of tumor spheroids: importance of dosage for penetration, and affinity for retention. Cancer Res. (2003) 63:1288–96. 12649189

[44] Schmidt MM Wittrup KD . A modeling analysis of the effects of molecular size and binding affinity on tumor targeting. Mol Cancer Ther. (2009) 8:2861–74. doi: 10.1158/1535-7163.mct-09-0195 19825804 PMC4078872

[45] Rudnick SI Lou J Shaller CC Tang Y Klein-Szanto AJ Weiner LM . Influence of affinity and antigen internalization on the uptake and penetration of anti-HER2 antibodies in solid tumors. Cancer Res. (2011) 71:2250–9. doi: 10.1158/0008-5472.can-10-2277 21406401 PMC3077882

[46] Thurber GM Zajic SC Wittrup KD . Theoretic criteria for antibody penetration into solid tumors and micrometastases. J Nucl Med. (2007) 48:995–9. doi: 10.2967/jnumed.106.037069 17504872

[47] Rhoden JJ Wittrup KD . Dose dependence of intratumoral perivascular distribution of monoclonal antibodies. J Pharm Sci. (2012) 101:860–7. doi: 10.1002/jps.22801 22057714 PMC3971539

[48] Lu G Nishio N van den Berg NS Martin BA Fakurnejad S van Keulen S . Co-administered antibody improves penetration of antibody-dye conjugate into human cancers with implications for antibody-drug conjugates. Nat Commun. (2020) 11:5667. doi: 10.1038/s41467-020-19498-y 33168818 PMC7652891

[49] Jauw YWS O'Donoghue JA Zijlstra JM Hoekstra OS Menke-van der Houven van Oordt CW Morschhauser F . (89)Zr-immuno-PET: toward a noninvasive clinical tool to measure target engagement of therapeutic antibodies *in vivo*. J Nucl Med. (2019) 60:1825–32. doi: 10.2967/jnumed.118.224568 31147401 PMC12079161

[50] Menke-van der Houven van Oordt CW McGeoch A Bergstrom M McSherry I Smith DA Cleveland M . Immuno-PET imaging to assess target engagement: experience from (89)Zr-anti-HER3 mAb (GSK2849330) in patients with solid tumors. J Nucl Med. (2019) 60:902–9. doi: 10.2967/jnumed.118.214726 30733323 PMC6604691

[51] Ogitani Y Hagihara K Oitate M Naito H Agatsuma T . Bystander killing effect of DS-8201a, a novel anti-human epidermal growth factor receptor 2 antibody-drug conjugate, in tumors with human epidermal growth factor receptor 2 heterogeneity. Cancer Sci. (2016) 107:1039–46. 10.1111/cas.12966 PMC4946713 27166974

[52] Ogitani Y Aida T Hagihara K Yamaguchi J Ishii C Harada N . DS-8201a, a novel HER2-targeting ADC with a novel DNA topoisomerase I inhibitor, demonstrates a promising antitumor efficacy with differentiation from T-DM1. Clin Cancer Res. (2016) 22:5097–108. doi: 10.1158/1078-0432.ccr-15-2822 27026201

[53] Khera E Dong S Huang H de Bever L van Delft FL Thurber GM . Cellular-resolution imaging of bystander payload tissue penetration from antibody-drug conjugates. Mol Cancer Ther. (2022) 21:310–21. doi: 10.1158/1535-7163.mct-21-0580 34911819 PMC8832507

[54] Wang Y Cheng X Li X Chen W Zhang X Liu Y . Bystander effect in antibody-drug conjugates: Navigating the fine line in tumor heterogeneity. Crit Rev Oncol Hematol. (2025) 216:104979. doi: 10.1016/j.critrevonc.2025.104979 41061840

[55] Wei Q Yang T Zhu J Zhang Z Yang L Zhang Y . Spatiotemporal quantification of HER2-targeting antibody-drug conjugate bystander activity and enhancement of solid tumor penetration. Clin Cancer Res. (2024) 30:984–97. doi: 10.1158/1078-0432.ccr-23-1725 38113039

[56] Cilliers C Guo H Liao J Christodolu N Thurber GM . Multiscale modeling of antibody-drug conjugates: Connecting tissue and cellular distribution to whole animal pharmacokinetics and potential implications for efficacy. AAPS J. (2016) 18:1117–30. doi: 10.1208/s12248-016-9940-z 27287046 PMC4996726

[57] Burton JK Bottino D Secomb TW . A systems pharmacology model for drug delivery to solid tumors by antibody-drug conjugates: Implications for bystander effects. AAPS J. (2019) 22:12. doi: 10.1208/s12248-019-0390-2 31828446 PMC7098807

[58] Singh AP Guo L Verma A Wong GG Thurber GM Shah DK . Antibody coadministration as a strategy to overcome binding-site barrier for ADCs: A quantitative investigation. AAPS J. (2020) 22:28. doi: 10.1208/s12248-019-0387-x 31938899 PMC8382310

[59] Bordeau BM Yang Y Balthasar JP . Transient competitive inhibition bypasses the binding site barrier to improve tumor penetration of trastuzumab and enhance T-DM1 efficacy. Cancer Res. (2021) 81:4145–54. doi: 10.1158/0008-5472.can-20-3822 33727230 PMC8338739

[60] van Dongen G . Improving tumor penetration of antibodies and antibody-drug conjugates: Taking away the barriers for Trojan horses. Cancer Res. (2021) 81:3956–7. doi: 10.1201/9781003573302-84 34341064

[61] Cilliers C Menezes B Nessler I Linderman J Thurber GM . Improved tumor penetration and single-cell targeting of antibody-drug conjugates increases anticancer efficacy and host survival. Cancer Res. (2018) 78:758–68. doi: 10.1158/0008-5472.can-17-1638 29217763 PMC5903206

[62] Morcos PN Li J Hosseini I Li CC . Quantitative clinical pharmacology of T-cell engaging bispecifics: Current perspectives and opportunities. Clin Transl Sci. (2021) 14:75–85. doi: 10.1111/cts.12877 32882099 PMC7877841

[63] Jauw YW Menke-van der Houven van Oordt CW Hoekstra OS Hendrikse NH Vugts DJ Zijlstra JM . Immuno-positron emission tomography with zirconium-89-labeled monoclonal antibodies in oncology: What can we learn from initial clinical trials? Front Pharmacol. (2016) 7:131. doi: 10.3389/fphar.2016.00131 27252651 PMC4877495

[64] Niemeijer AN Leung D Huisman MC Bahce I Hoekstra OS van Dongen G . Whole body PD-1 and PD-L1 positron emission tomography in patients with non-small-cell lung cancer. Nat Commun. (2018) 9:4664. doi: 10.1038/s41467-018-07131-y 30405135 PMC6220188

[65] Kok IC Hooiveld JS van de Donk PP Giesen D van der Veen EL Lub-de Hooge MN . (89)Zr-pembrolizumab imaging as a non-invasive approach to assess clinical response to PD-1 blockade in cancer. Ann Oncol. (2022) 33:80–8. doi: 10.1016/j.annonc.2021.10.213 34736925

[66] Wijngaarden JE Jauw YWS Zwezerijnen GJC de Wit-van der Veen BJ Vugts DJ Zijlstra JM . Non-specific irreversible (89)Zr-mAb uptake in tumours: Evidence from biopsy-proven target-negative tumours using (89)Zr-immuno-PET. EJNMMI Res. (2024) 14:18. doi: 10.1186/s13550-024-01079-5 38358425 PMC10869322

[67] Huisman MC Pouw JEE Golla SSV Huglo D Morschhauser F Zijlstra JM . (89)Zr-mAb uptake interpretation requires the use of tissue to plasma ratios corrected for antibody catabolism. EJNMMI Res. (2025) 15:122. doi: 10.70534/rvln6580 PMC12474831 41003920

[68] Mohr P van Sluis J Lub-de Hooge MN Lammertsma AA Brouwers AH Tsoumpas C . Advances and challenges in immunoPET methodology. Front Nucl Med. (2024) 4:1360710. doi: 10.3389/fnume.2024.1360710 39355220 PMC11440922

[69] van Dongen G Beaino W Windhorst AD Zwezerijnen GJC Oprea-Lager DE Hendrikse NH . The role of (89)Zr-immuno-PET in navigating and derisking the development of biopharmaceuticals. J Nucl Med. (2021) 62:438–45. doi: 10.2967/jnumed.119.239558 33277395

[70] Carmon KS Azhdarinia A . Application of immuno-PET in antibody-drug conjugate development. Mol Imaging. (2018) 17:1536012118801223. doi: 10.1177/1536012118801223 30370812 PMC6207972

[71] McKnight BN Viola-Villegas NT . (89) Zr-immunoPET companion diagnostics and their impact in clinical drug development. J Labelled Comp Radiopharm. (2018) 61:727–38. doi: 10.1002/jlcr.3605 29341222 PMC6050145

[72] Shen JX Dodge R Oberstein S Cunliffe J Ramani V Mohana Sundaram S . Updated recommendations for the bioanalysis of antibody-drug conjugates (ADC) from the ADC working group of the AAPS Bioanalytical Community. AAPS J. (2026) 28:121. doi: 10.1208/s12248-026-01268-1 42387157

[73] Rediti M Fimereli D Mileva M Wimana Z Venet D Flamen P . Integrating molecular imaging and transcriptomic profiling in advanced HER2-positive breast cancer receiving trastuzumab emtansine: An analysis of the ZEPHIR clinical trial. Clin Cancer Res. (2025) 31:110–21. doi: 10.1158/1078-0432.ccr-24-1007 39470686

[74] Rosenthal EL Warram JM de Boer E Chung TK Korb ML Brandwein-Gensler M . Safety and tumor specificity of cetuximab-IRDye800 for surgical navigation in head and neck cancer. Clin Cancer Res. (2015) 21:3658–66. doi: 10.1158/1078-0432.ccr-14-3284 25904751 PMC4909371

[75] Lamberts LE Koch M de Jong JS Adams ALL Glatz J Kranendonk MEG . Tumor-specific uptake of fluorescent bevacizumab-IRDye800CW microdosing in patients with primary breast cancer: A phase I feasibility study. Clin Cancer Res. (2017) 23:2730–41. doi: 10.1158/1078-0432.ccr-16-0437 28119364

[76] Lu G van den Berg NS Martin BA Nishio N Hart ZP van Keulen S . Tumour-specific fluorescence-guided surgery for pancreatic cancer using panitumumab-IRDye800CW: A phase 1 single-centre, open-label, single-arm, dose-escalation study. Lancet Gastroenterol Hepatol. (2020) 5:753–64. doi: 10.1016/s2468-1253(20)30088-1 32416764 PMC7367758

[77] Zhou Q van den Berg NS Kang W Pei J Nishio N van Keulen S . Factors for differential outcome across cancers in clinical molecule-targeted fluorescence imaging. J Nucl Med. (2022) 63:1693–700. doi: 10.2967/jnumed.121.263674 35332092 PMC9635681

[78] Moore LS Rosenthal EL de Boer E Prince AC Patel N Richman JM . Effects of an unlabeled loading dose on tumor-specific uptake of a fluorescently labeled antibody for optical surgical navigation. Mol Imaging Biol. (2017) 19:610–6. doi: 10.1007/s11307-016-1022-1 27830425 PMC5679006

[79] Li M Zhao X Yu C Wang L . Antibody-drug conjugate overview: A state-of-the-art manufacturing process and control strategy. Pharm Res. (2024) 41:419–40. doi: 10.1007/s11095-023-03649-z 38366236

[80] Hu Z Liao Y Niu M Kong Z Vankelecom H Cheng J . Emerging organoids and organoids-on-chip platforms for translational development of antibody–drug conjugates and next-generation bioconjugates. Acta Pharm Sin B. (2026). doi: 10.1016/j.apsb.2026.05.032 42764940 PMC13589940

