# Supplementary material for: Spatial PK–PD mismatch in tumor-targeting antibodies and ADCs: from lesion access to pharmacologic execution
Source: Front Oncol. 2026 Sep 22;16:1952445. doi: 10.3389/fonc.2026.1952445 (PMC13639573; doi:10.3389/fonc.2026.1952445)
Supplement: Supplementary file 1 [file Table1.docx]

**Supplementary material for: Spatial PK–PD mismatch in tumor-targeting antibodies and ADCs: from lesion access to pharmacologic execution**

**Supplementary Table S1. Full technical fields for direct human administered-drug and lesion-resolved exposure–outcome studies.**

Fields were transcribed from the cited primary reports and linked trial records. “Not reported” is used only when the source did not provide the field; no unreported value was inferred.

| **Study / tier** | **Tumor and sample** | **Construct relationship** | **Dose / antibody mass** | **Timing** | **Spatial method** | **Principal result / boundary** |
| --- | --- | --- | --- | --- | --- | --- |
| {Welt, 1990} Tier 1 | Metastatic colorectal cancer with hepatic metastases Treated N=20 Evaluable N=20 for localization (selective localization in 19/20) | Radiolabeled target-directed antibody; not a clinically administered therapeutic analogue | 131I-mAb A33, 2–5 mCi and 0.2–50 mg protein, IV | 7–8 days before surgery | Whole-body imaging, lesion localization, and quantitative tissue analysis | Selective but variable tumor localization; no cell-level target-occupancy measurement |
| {Scott, 2005} Tier 1 | Colorectal carcinoma Treated N=12 Evaluable N=12 | Radiolabeled humanized target-directed antibody | 400 MBq 131I-huA33 plus 40 MBq 125I-huA33; protein 0.25, 1, 5, or 10 mg/m², IV | 7 days before surgery; serial imaging and tissue sampling | Plasma PK, whole-body/lesion imaging, and quantitative tissue disposition | Sustained target-associated tumor uptake with interpatient and intratumoral variability |
| {Ciprotti, 2014} Tier 1 | Colorectal carcinoma Treated N=12 Evaluable N=12 | Radiolabeled humanized target-directed antibody; resection substudy of the A33 phase I program | 131I-huA33; phase I protein-dose range 0.25–10 mg/m², IV | 7 days before surgery | Macroscopic and microscopic autoradiography with kinetic tissue analysis | Heterogeneous and time-dependent microdistribution; radioactivity does not prove intact-antibody occupancy |
| {Dijkers, 2010} Tier 1 | Metastatic HER2-positive breast cancer Treated N=14 Evaluable N=14 | Radiolabeled therapeutic analogue of trastuzumab | 37 MBq 89Zr-trastuzumab; 10 or 50 mg in trastuzumab-naive patients and 10 mg in patients receiving trastuzumab | PET on days 2–5; optimal visualization on days 4–5 | Whole-body immunoPET and lesion uptake quantification | Marked interlesional heterogeneity; tracer mass and residualizing signal influence interpretation |
| {Rosenthal, 2015} Tier 1 | Head and neck squamous cell carcinoma Treated N=12 Evaluable N=12 | Fluorescently labeled therapeutic analogue of cetuximab | Cetuximab–IRDye800, 2.5, 25, or 62.5 mg/m², IV | Infusion on day 0; surgery on day 3 | In vivo, intraoperative, specimen, and tissue fluorescence imaging | Feasible tumor-selective imaging across scales; early safety/specificity study without efficacy testing |
| {Lamberts, 2017} Tier 1 | Primary breast cancer Treated N=20 Evaluable N=20 | Fluorescent anti-VEGF antibody microdose | Bevacizumab–IRDye800CW, 4.5 mg IV | 3 days before surgery | In vivo and ex vivo fluorescence imaging with pathology correlation | Tumor-specific microdose uptake was measurable; vascular target and resectable population limit generalization |
| {Moore, 2017} Tier 1 | Head and neck squamous cell carcinoma Treated N=6 Evaluable N=6 (3 per preload cohort) | Fluorescently labeled therapeutic analogue with unlabeled parent-antibody preload | Unlabeled cetuximab 10 or 100 mg, followed 1 h later by cetuximab–IRDye800CW 25 mg/m², IV | Surgery 3–4 days after labeled-antibody infusion | Plasma PK, in vivo optical imaging, and specimen fluorescence | The 100-mg preload increased circulating labeled antibody, tumor fluorescence, and tumor-to-background ratio; very small cohort |
| {Menke-van der Houven van Oordt, 2019} Tier 1 | Advanced HER3-positive solid tumors Treated N=6 Evaluable N=6 | Radiolabeled therapeutic analogue of anti-HER3 antibody GSK2849330 | 37 MBq 89Zr-GSK2849330; baseline total mass 8 or 24 mg; repeat study after graded total masses from 24 mg to 30 mg/kg | PET on days 2 and 5; repeat administration approximately 2 weeks later | Whole-body immunoPET with target-engagement and mass-saturation modeling | Demonstrated imaging-supported antibody-mass selection and target saturation; six-patient development study |
| {Lu, 2020a} Tier 1 | Resectable head and neck squamous cell carcinoma Treated N=24 Evaluable N=22 in the key loading-dose microdistribution comparison (12 loading; 10 non-loading) | Fluorescently labeled therapeutic analogue with or without unlabeled panitumumab | Loading cohort: 100 mg panitumumab then 0.5 or 1 mg/kg panitumumab–IRDye800CW; non-loading cohort: 25 or 50 mg labeled antibody; total antibody 0.3–2.6 mg/kg | 1–5 days before surgery | Quantitative tumor concentration, whole-slide fluorescence, and vessel-distance analysis | Coadministration broadened microscopic distribution without increasing normal-tissue uptake; no therapeutic-outcome endpoint |
| {Lu, 2020b} Tier 1 | Resectable head and neck squamous cell carcinoma Treated N=24 Evaluable N=24 for imaging analyses; fresh-tissue concentration available in 22 | Fluorescently labeled therapeutic analogue of panitumumab | Panitumumab–IRDye800CW, 25–95 mg IV | Several days before surgery (trial window 1–5 days) | Tumor concentration, multiscale fluorescence, vessel-distance analysis, and pretreatment MRI features | Quantified interpatient, intertumor, and intratumor delivery heterogeneity; tumor size and architecture predicted delivery |
| {Lu, 2026} Tier 1 | HNSCC, pancreatic ductal adenocarcinoma, and NSCLC Treated N=50 enrolled across three phase I trials Evaluable N=Bulk quantification: 43 (HNSCC 25; NSCLC 4; PDAC 14); spatial cohort: 30 (HNSCC 18; PDAC 12) | Fluorescently labeled therapeutic analogue of panitumumab at subtherapeutic imaging doses | HNSCC: 50 mg; PDAC: 25/50/75 mg with 100 mg unlabeled panitumumab or 50 mg alone; NSCLC: 25/50/75 mg | HNSCC/NSCLC 1–5 days and PDAC 2–5 days before surgery | Bulk tumor quantification integrated with single-cell drug imaging and high-plex spatial proteomics | Directly mapped heterogeneous cellular delivery and engagement; periostin-rich ECM and FAP-positive CAF neighborhoods were associated with low delivery |
| {Gebhart, 2016} Tier 2 | Advanced HER2-positive breast cancer Treated N=60 enrolled Evaluable N=56 analyzed | Radiolabeled therapeutic analogue used to characterize accessible HER2 before T-DM1 | 37 MBq (±10%) 89Zr-trastuzumab plus 50 mg unlabeled trastuzumab; T-DM1 3.6 mg/kg every 3 weeks | HER2-PET 4 days after tracer; early FDG-PET before cycle 2; response after 3 cycles | Whole-body lesion-resolved HER2-PET integrated with early FDG-PET and treatment outcome | Negative or heterogeneous HER2-PET patterns identified high early-failure risk; nonrandomized predictive evidence |
| {Mortimer, 2022} Tier 2 | Metastatic HER2-positive breast cancer Treated N=10 Evaluable N=10 | Radiolabeled therapeutic analogue used before T-DM1 | 45 mg unlabeled trastuzumab followed by 5 mg 64Cu-DOTA-trastuzumab; T-DM1 3.6 mg/kg every 3 weeks | PET at 21–25 and 47–49 h after tracer, before T-DM1 | Lesion-resolved PET uptake associated with time to treatment failure | Baseline uptake showed exploratory association with T-DM1 outcome; ten-patient pilot |
| {Bensch, 2018a} Tier 2 | Advanced NSCLC, bladder cancer, and triple-negative breast cancer Treated N=22 Evaluable N=22 | Radiolabeled therapeutic analogue of atezolizumab; immune-checkpoint boundary case | 10 mg unlabeled atezolizumab followed by 37 MBq 89Zr-atezolizumab (approximately 1 mg); subsequent atezolizumab 1,200 mg every 3 weeks | PET at 1 h and days 2, 4, and 7; biopsy 8–10 days after tracer | Whole-body uptake, tissue PD-L1/immune context, and response association | Higher uptake was associated with response; immune-target boundary evidence, not tumor-cell-targeting ADC pharmacology |
| {Kok, 2022} Tier 2 | Advanced melanoma or NSCLC Treated N=18 Evaluable N=18 | Radiolabeled therapeutic analogue of pembrolizumab; immune-checkpoint boundary case | 37 MBq 89Zr-pembrolizumab (approximately 2.5 mg) plus unlabeled antibody to total mass 5 or 10 mg | PET on days 2, 4, and 7 before pembrolizumab treatment | Whole-body biodistribution and exploratory uptake–outcome association | Day-7 imaging at 5-mg total mass was most informative; not directly generalizable to tumor-cell-targeting ADCs |
| {Rediti, 2025} Tier 2 | ZEPHIR molecular-imaging substudy in HER2-positive metastatic breast cancer Treated N=Parent ZEPHIR cohort 60 Evaluable N=24 matched biopsied metastases | Parent ZEPHIR 89Zr-trastuzumab exposure linked to lesion transcriptomics | Parent protocol: 37 MBq (±10%) 89Zr-trastuzumab plus 50 mg unlabeled trastuzumab before T-DM1 | Baseline HER2-PET and early FDG-PET; biopsy-linked molecular analysis | Lesion-resolved imaging integrated with matched metastasis transcriptomics | Low uptake and nonresponse were associated with extracellular-matrix and hypoxia-related programs; associative, not interventional |
